# ATP7A-fibulin-4 complex delivers copper in the Golgi to activate LOX in renal fibrosis

**DOI:** 10.1172/jci.insight.199028

**Published:** 2026-05-08

**Authors:** Wenqian Zhou, Yan Zheng, Yuqing Liu, Jing Liu, Yiguo Liu, Yangyang Niu, Ying Yu, Xiaoqin Zhang, Yingying Zhang, Chen Yu

**Affiliations:** Department of Nephrology, Tongji Hospital, School of Medicine, Tongji University, Shanghai, China

**Keywords:** Cell biology, Nephrology, Fibrosis

## Abstract

Lysyl oxidase (LOX) is a copper-dependent monoamine oxidase whose primary function is the covalent cross-linking of collagen and elastin in the extracellular matrix (ECM). However, the regulation of LOX activity in renal fibrosis is not well understood. Here, our study showed that (a) LOX expression and ECM cross-linking were markedly increased in fibrotic kidneys. Reduction of copper levels in the Golgi apparatus by treatment with the copper chelator tetrathiomolybdate or by specific knockdown of copper transporter 1 (CTR1) decreased LOX activity and ameliorated renal fibrosis. (b) Overexpression of ATP7A caused an elevation of copper ions within the Golgi apparatus, resulting in increased LOX activity and enhanced ECM crosslinking, thereby promoting the progression of renal fibrosis. Knockdown of ATP7A showed the opposite result. (c) FBLN4 was essential for the ATP7A-mediated transfer of copper to LOX and formed a ternary complex of ATP7A-FBLN4-LOX. Our research revealed that high ATP7A expression induced copper overload in the Golgi apparatuses. FBLN4 then assisted ATP7A in transporting this excess copper to LOX, resulting in LOX overactivation. This, in turn, catalyzed the cross-linking of ECM components, thereby accelerating renal fibrosis.

## Abstract 2

ATP7A regulates LOX activity through the induction of intra-Golgi copper accumulation, and this process is involved in FBLN4-mediated the interaction of ATP7A with LOX

## Graphical abstract

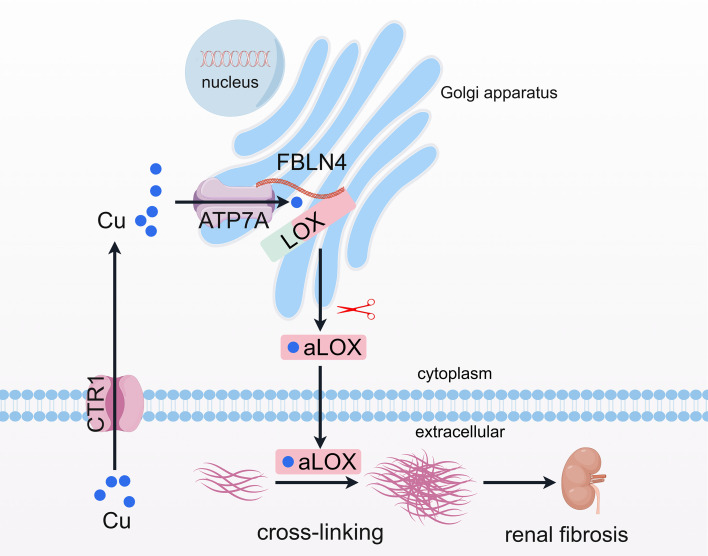

## Introduction

Renal fibrosis is characterized by the excessive accumulation of extracellular matrix (ECM) components within the kidney (1). This accumulation results from a disruption in the normal balance between ECM deposition and degradation, which are 2 key processes during the progression of fibrotic diseases (2, 3). Excessive ECM cross-linking in renal fibrosis contributes to a reduction of ECM degradation, yet research in this area remains limited.

Lysyl oxidase (LOX) is an amine oxidase that plays a critical role in catalyzing the covalent cross-linking of collagen and elastin to form insoluble ECM components (4). Our and other groups have demonstrated that LOX serves as a key mediator in promoting renal fibrosis (5–7). Targeting LOX has been shown to significantly ameliorate fibrotic progression. Mechanistically, LOX catalyzes the oxidative deamination of lysine and hydroxylysine residues in collagen and elastin, which is the initiation step of the formation of ECM. Inhibition of LOX enzymatic activity may effectively interfere with its binding to the substrate (collagen and elastin), thereby suggesting LOX activity as a more promising therapeutic target for renal fibrosis (5, 6). However, the underlying regulatory mechanisms of LOX activity in renal fibrosis remain unclear.

LOX process an active enzyme domain at the C-terminal region that includes conserved copper binding residues and a conserved tyrosine and lysine residue that become the lysyl tyrosyl quinone (LTQ) cofactor required for enzyme activity (8, 9). Notably, copper plays a crucial role in the biosynthesis of LTQ as well as in maintaining the structural integrity of both cofactors and the protein structure (10–12). Therefore, copper is an essential cofactor necessary for LOX to perform its enzymatic function. Our previous studies have demonstrated that copper ions accumulate intracellularly in renal fibrosis. Additionally, we observed that copper chelation effectively inhibits the progression of renal fibrosis (6). LOX is activated by copper within the Golgi apparatus (13). However, to our knowledge, no studies to date have elucidated the regulatory mechanisms governing copper homeostasis in the Golgi apparatus and its association with the progression of renal fibrosis.

In this study, we investigated the role of LOX activity and its regulatory mechanisms at the subcellular level during renal fibrosis and addressed the following three central questions. (a) How does LOX activity contribute to renal fibrosis initiation and progression, and is Golgi copper accumulation a key activating mechanism? (b) What drives intracellular copper accumulation specifically within the Golgi apparatus? (c) Could inhibiting copper delivery to the Golgi represent a viable therapeutic strategy for renal fibrosis? Our findings provide mechanistic insight into Golgi copper-mediated LOX activation in fibrosis and highlight a potential targeted treatment approach.

## Results

### Increased LOX activity promotes ECM cross-linking in fibrotic kidneys.

**Figure 1:**
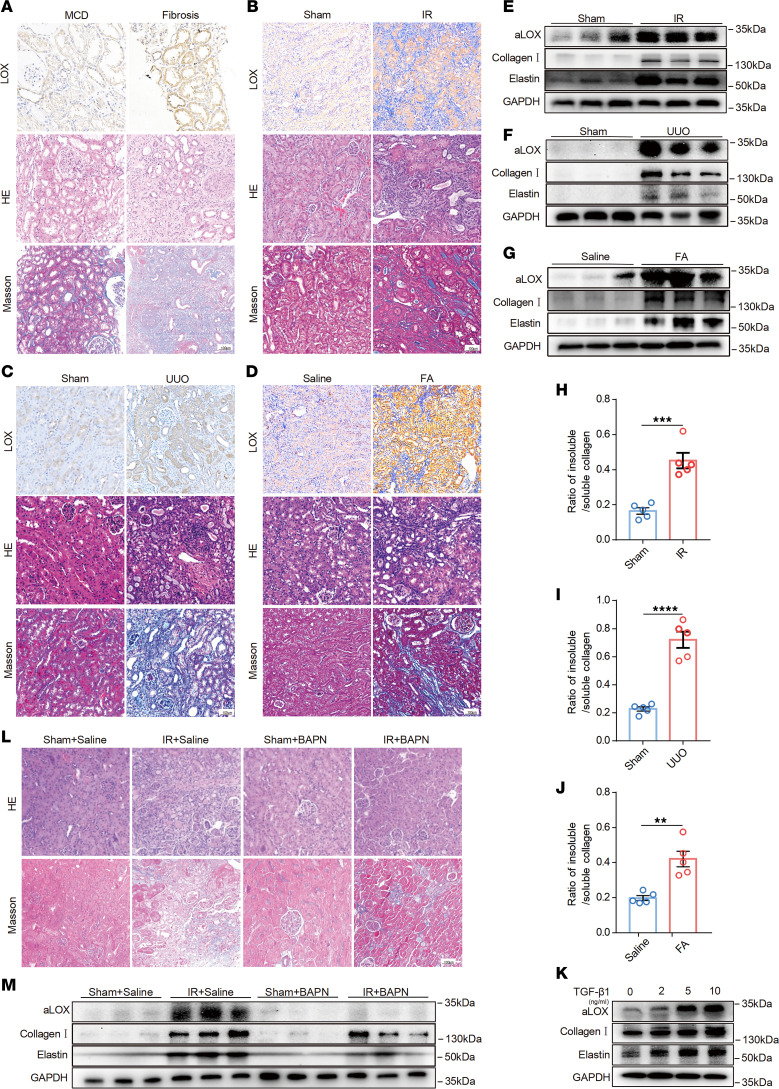
Expression of LOX and ECM cross-linking were markedly increased in fibrotic kidneys. (**A**) Representative images of immunohistochemical staining with LOX antibody, H&E, and Masson’s trichrome in kidney sections from patients with and without minimal change disease (MCD) group renal fibrosis (*n* = 5). Original magnification, ×200. Scale bar: 100 μm. (**B**–**D**) Representative images of immunohistochemical staining of LOX, H&E, and Masson’s trichrome in kidney sections from renal fibrosis models induced by IR, UUO, and FA (*n* = 5). Original magnification, ×200. Scale bar: 100 μm. (**E**–**G**) Western immunoblots of activated aLOX (aLOX), collagen I, and elastin levels in kidneys collected from mouse models induced by IR, UUO, and FA (*n* = 5). (**H**–**J**) The ratio of insoluble/soluble collagen was analyzed in kidneys collected from mouse models induced by IR, UUO, and FA (*n* = 5). (**K**) Western immunoblots of the expression of aLOX, collagen I, and elastin levels in NRK-52E cells treated with TGF-β1 (*n* = 3). For BAPN treatment, 4 groups (*n* = 5/each group) of C57BL/6 mice were prepared: sham + saline; sham + BAPN (100 mg/kg/d); IR + saline; and IR + BAPN. (**L**) Representative images of H&E and Masson’s trichrome staining of kidney sections. Original magnification, ×200. Scale bar: 100 μm. (**M**) Western immunoblots of aLOX, collagen I, and elastin levels in mouse kidneys. Data are shown as the mean ± SEM. Statistics used included a 2-tailed *t* test. **P < 0.01, ***P < 0.001, ****P < 0.0001. IR, ischemia/reperfusion injury; FA, folic acid; UUO, unilateral urethral obstruction.

LOX catalyzes the covalent cross-linking of soluble collagens and elastin into insoluble and mature fibers (10). To identify the role of LOX in renal fibrosis, we first assessed the level of total LOX in human kidney biopsy tissues of patients by immunohistochemistry staining. Compared with the minimal change disease (MCD) group, increased LOX expression was observed in both tubular cells and interstitial tissue in fibrotic kidneys (Figure 1A and Supplemental Figure 1A; supplemental material available online with this article; https://doi.org/10.1172/jci.insight.199028DS1). LOX refers to the full-length protein, which lacks intrinsic catalytic activity. Upon proteolytic cleavage, the C-terminal fragment containing the lysine tyrosylquinone (LTQ) domain becomes enzymatically active in the presence of copper ions. LOX contributes to fibrosis through 2 steps: upregulated expression, followed by proteolytic activation. In order to explore the role of the LOX activity under the fibrotic condition, environment, the active LOX domains (aLOX) were detected by Western blot in the mouse kidney tissues from renal fibrosis models induced by ischemia/reperfusion (IR), unilateral urethral obstruction (UUO), and folic acid (FA) (Figure 1, B–D, and Supplemental Figure 1, B–D) (5). We found that aLOX, collagen I, and elastin were also markedly increased in kidneys with IR, UUO, and FA injury, compared with sham-operated kidneys, as determined by Western blotting (Figure 1, E–G, and Supplemental Figure 1, E–G).

Given the characteristic activity of LOX, a higher ratio of insoluble to soluble collagen indicated increased LOX activity. Furthermore, the ratios of insoluble collagen to soluble collagen in the IR, UUO, and FA groups were significantly higher than those in the sham-operated groups (Figure 1, H–J). Based on the subcellular localization of LOX, rat renal tubular epithelial cells (NRK-52E) were selected for in vitro experiments. aLOX expression, collagen I, and elastin were also upregulated in NRK-52E cells following treatment with TGF-β1 (Figure 1K and Supplemental Figure 1H).

β-Aminopropionitrile (BAPN) acts as a specific inhibitor of LOX activity by targeting the LTQ cofactor located within the catalytic and ubiquitination domains of the enzyme (14). To further assess the role of LOX activity in renal fibrosis, we examined the effect of BAPN on IR- and UUO-induced injury in mouse kidneys. We found that ECM proteins were extensively deposited within the interstitial spaces of either IR- or UUO-injured kidneys, whereas administration of BAPN significantly reduced collagen deposition (Figure 1, L and M, and Supplemental Figures 1 and 2). These results indicate that LOX expression and LOX activity are significantly upregulated in fibrotic kidneys and that suppression of LOX activity effectively attenuates ECM cross-linking and ameliorates renal fibrosis.

### Reducing copper levels in Golgi apparatus can reduce LOX activity and improve renal fibrosis.

**Figure 2:**
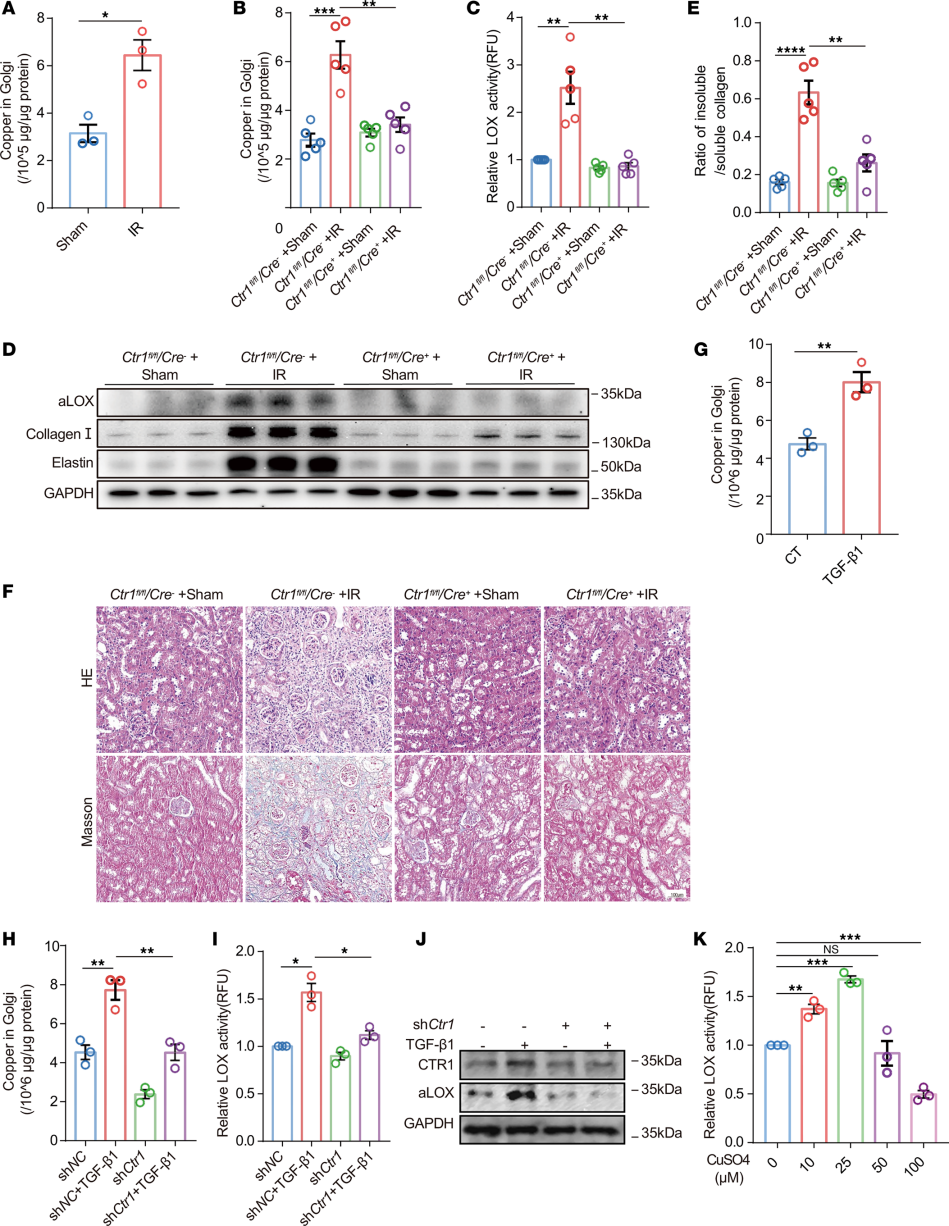
Reducing Golgi copper ion levels by downregulation of CTR1 decreases LOX activity and alleviates renal fibrosis. (**A**) The copper level was detected in the Golgi apparatus of kidney tissue collected from an IR-induced mouse model (*n* = 3). For the analyses of *Ctr1*-manipulated mice, *Ctr1*^fl/fl^/*Cre*^–^ and *Ctr1*^fl/fl^/*Cre*^+^ mice were randomly divided into the following 4 groups (*n* = 5/each group): *Ctr1*^fl/fl^/*Cre*^–^ mice + sham; *Ctr1*^fl/fl^/*Cre*^–^ mice + IR; *Ctr1*^fl/fl^/*Cre*^+^ mice + sham; and *Ctr1*^fl/fl^/*Cre*^+^ mice + IR (**B**–**F**). (**B**) Inductively coupled plasma mass spectrometry (ICP-MS) analysis of copper concentrations of Golgi apparatus in kidney tissues. (**C**) LOX activity in kidney tissues was determined using the Fluorometric Lysyl Oxidase Assay Kit. (**D**) Western immunoblots of the expression of aLOX, collagen I, and elastin levels in mouse kidneys. (**E**) The ratio of insoluble/soluble collagen was analyzed in kidneys collected from IR-induced mice models. (**F**) Representative images of H&E and Masson’s trichrome staining of kidney sections. Original magnification, ×200. Scale bar: 100 μm. (**G**) The copper level was detected in the Golgi apparatus of NRK-52E cells treated with or without TGF-β1 (*n* = 3). (**H**) The copper level was detected in the Golgi apparatus of NRK-52E cells treated with TGF-β1 after downregulation of *Ctr1*(*n* = 3). (**I**) LOX activity was determined in the culture medium of NRK-52E cells treated with TGF-β1 after downregulation of *Ctr1* using the Fluorometric Lysyl Oxidase Assay Kit (*n* = 3). (**J**) Western immunoblots analysis of aLOX and CTR1 in NRK-52E cells treated with TGF-β1 after downregulation of *Ctr1* (*n* = 3). (**K**) LOX activity in NRK-52E cells treated with multiple concentrations of copper sulfate was determined using the Fluorometric Lysyl Oxidase Assay Kit (*n* = 3). Data are shown as the mean ± SEM. Statistics used included a 2-tailed *t* test (2 groups, in **A** and **G**) or 1-way ANOVA (multiple groups, in **B**, **C**, **E**, **H**, **I**, and **K**). *P < 0.05, **P < 0.01, ***P < 0.001, ****P < 0.0001.

Previous studies reported that LOX is activated in the Golgi apparatus, and copper is an essential metal ion for the activation of LOX (10). We found elevated copper levels in the Golgi apparatus extracted from fibrosis kidney tissues induced by IR in vivo as well as in tubule cells treated with TGF-β1 in vitro (Figure 2, A and G). In our previous study, we revealed that copper was significantly elevated in kidney fibrotic tissues, and this increase was mediated by the upregulation of copper transporter 1 (CTR1), a copper uptake transporter (6). CTR1 is mainly located in the renal tubular epithelial cells of the kidney, according to the analysis (RBK RID, 14-4KBC; Healthy Mouse Database, ref. 15; https://humphreyslab.com/SingleCell/) of kidney single-cell sequencing database and revealed high CTR1 expression in proximal tubular epithelial cells (Supplemental Figure 3A) (15). Immunofluorescence confirmed CTR1 colocalization with the proximal tubule marker aquaporin-1 (AQP1) but not with distal convoluted tubule markers (Supplemental Figure 3B). Next, we generated *Ctr1^fl/fl^* mice and crossed them with *Ggt1-Cre* mice to specifically knock-down *Ctr1* expression in renal tubule epithelial cells (Supplemental Figure 3C). In renal tubular epithelial cells, *Ctr1* knockout significantly reduced intracellular copper levels, including Golgi apparatus, in fibrotic renal tissues (Figure 2B). Fluorometric Lysyl Oxidase Assay and Western blot assay showed that renal LOX activity was downregulated following *Ctr1* downregulation in fibrotic kidneys (Figure 2, C–E, and Supplemental Figure 4A). H&E staining and Masson’s trichrome staining revealed that the degree of tubular atrophy, dilatation, and collagen deposition induced by IR injury was markedly ameliorated in *Ctr1^fl/fl^*/*Ggt1-Cre^+^* mice (Figure 2F and Supplemental Figure 4B). Downregulation of *Ctr1* in *Ctr1^fl/fl^*/*Ggt1-Cre^+^* mice also reduced protein levels of fibrotic markers, including collagen I and elastin (Figure 2D and Supplemental Figure 4A). To validate the relationship between LOX activity and copper ions, NRK-52E cells were treated with different concentrations of copper sulfate (CuSO_4_). Enzymatic assays revealed that LOX activity peaked at 25 μM CuSO_4_, started to decline at 50 μM, and was notably reduced compared with controls at 100 μM, indicating that copper ions promote LOX activation within an optimal concentration range (Figure 2K). Additionally, downregulation of *Ctr1* significantly reduced the TGF-β1–induced increase in Golgi copper concentration and LOX activity (Figure 2, G–J, and Supplemental Figure 4C). Taken together, these results suggest that reducing renal Golgi copper by knocking down *Ctr1* can effectively attenuates renal LOX activity and alleviates renal fibrosis.

**Figure 3:**
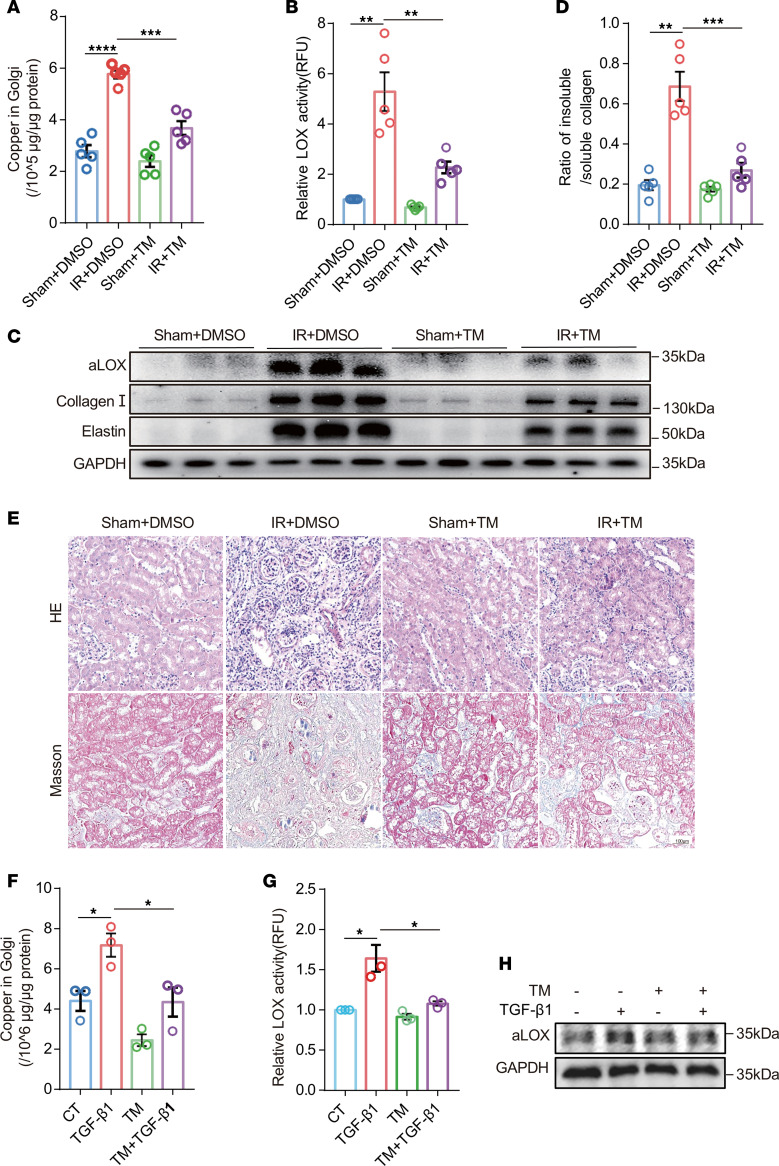
Reducing copper in Golgi apparatus with Cu chelators mitigated renal fibrosis via reducing LOX activity. For TM treatment, 4 groups of C57BL/6 mice were prepared (*n* = 5/each group): sham + DMSO; sham + TM (30 mg/kg); IR + DMSO; and IR + TM (**A**–**E**). (**A**) ICP-MS analysis of copper concentrations of Golgi apparatus in kidney tissues. (**B**) LOX activity in kidney tissues was determined using the Fluorometric Lysyl Oxidase Assay Kit. (**C**) Western immunoblots analysis of aLOX, collagen I, and elastin levels in mouse kidneys. (**D**) The ratio of insoluble/soluble collagen was analyzed in kidneys collected from IR-induced mouse models. (**E**) Representative images of H&E and Masson’s trichrome staining of kidney sections. Original magnification, ×200. Scale bar: 100 μm. (**F**) The copper level was detected in the Golgi apparatus of NRK-52E cells treated with or without (CT) TGF-β1 or TM (*n* = 3). (**G**) LOX activity was determined in the culture medium of NRK-52E cells treated with or without TM after stimulated with TGF-β1 using the Fluorometric Lysyl Oxidase Assay Kit (*n* = 3). (**H**) Western immunoblot analysis of aLOX in NRK-52E cells treated with or without TM after stimulated with TGF-β1 (*n* = 3). Data are shown as the mean ± SEM. Statistics used included 1-way ANOVA. *P < 0.05, **P < 0.01, ***P < 0.001, ****P < 0.0001.

Tetrathiomolybdate (TM), a copper chelating agent, chelates copper both intracellularly and extracellularly. We found that TM could reduce the IR-induced elevation of copper in the Golgi apparatus by pharmacological inhibition (Figure 3A). Compared with IR mice without TM treatment, TM treatment significantly ameliorated renal level of LOX activity (Figure 3, B and C, and Supplemental Figure 4D) and reduced the ratio of insoluble collagen to soluble collagen (Figure 3D). TM treatment alleviated IR-induced renal fibrosis, as shown by H&E and Masson staining (Figure 3E and Supplemental Figure 4E), and reduced collagen I and elastin expression in TM-treated mice compared with that in the sham group (Figure 3C and Supplemental Figure 4D). Consistent with in vivo findings, we found that in NRK-52E cells treated with TGF-β1, TM treatment resulted in lower Golgi copper concentration and recovered LOX activity (Figure 3, F–H, and Supplemental Figure 4F). These findings highlight the key role of Golgi copper ions in LOX activation.

### ATP7A, but not ATP7B, modulates LOX activity through promoting Golgi copper overloads in renal fibrosis.

**Figure 4:**
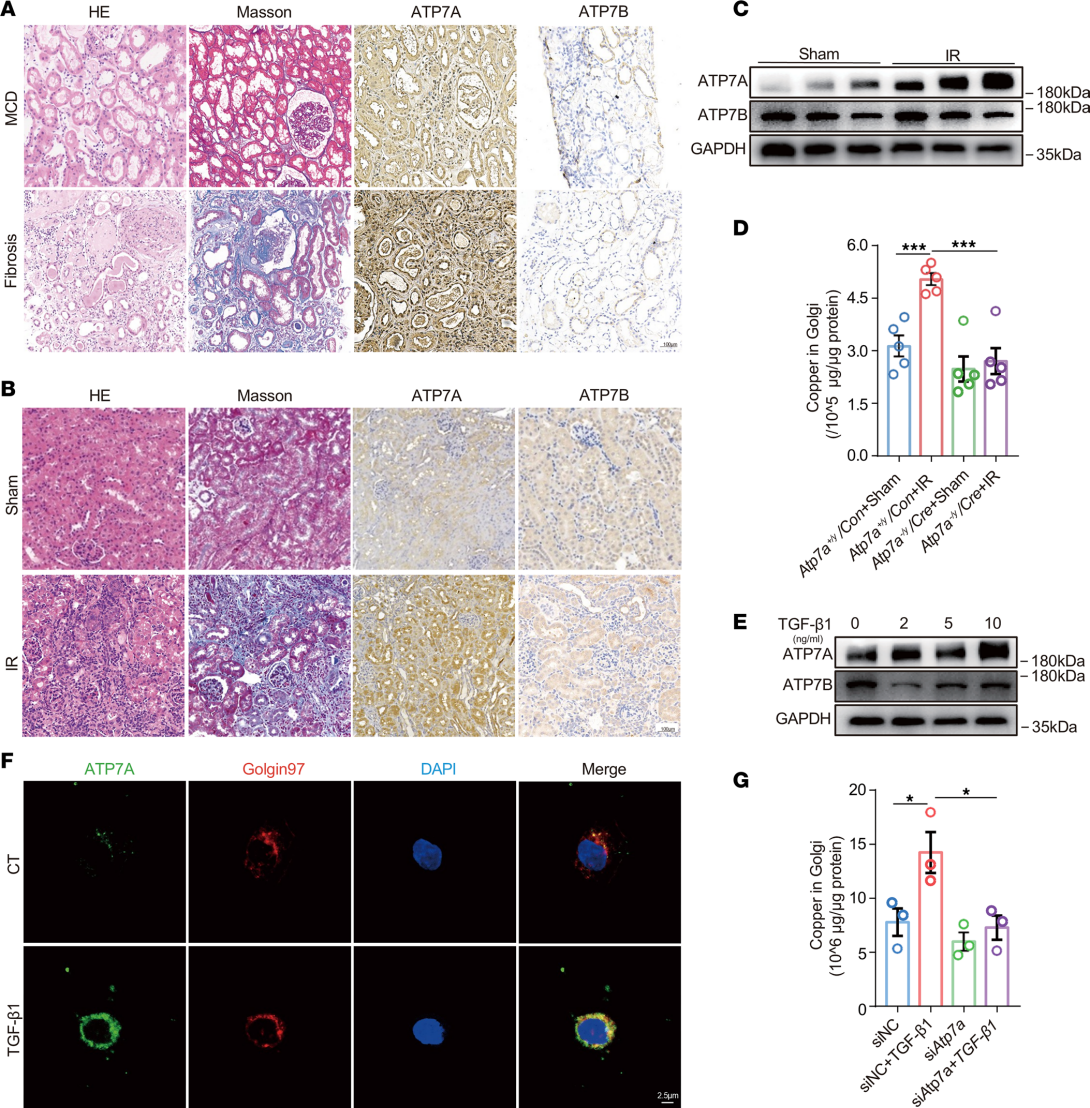
ATP7A mediates the increase of Golgi copper level in renal fibrosis. (**A**) Representative images of immunohistochemical staining with ATP7A, ATP7B, H&E, and Masson’s trichrome in kidney sections from patients with and without renal fibrosis (*n* = 5). Original magnification, ×200. Scale bar: 100 μm. (**B**) Representative images of immunohistochemical staining of ATP7A, ATP7B, H&E, and Masson’s trichrome in kidney sections from renal fibrosis models induced by IR (*n* = 5). Original magnification, ×200. Scale bar: 100 μm. (**C**) Western immunoblots analysis of ATP7A and ATP7B expression in mouse kidneys (*n* = 5). (**D**) The copper level was detected in the Golgi apparatus of kidneys collected from IR-induced mice with or without *Atp7a* downregulation (*n* = 5). (**E**) Western immunoblots analysis of ATP7A and ATP7B expression in NRK-52E cells treated with TGF-β1 (*n* = 3). (**F**) The colocalization of ATP7A with the Golgi apparatus was analyzed by immunofluorescent costaining in NRK-52E cells without (CT) or with TGF-β1 treatment (*n* = 3). Original magnification, ×1,000. Scale bar: 2.5 μm. (**G**) The copper level was detected in the Golgi apparatus of NRK-52E cells treated with TGF-β1 after downregulation of *Atp7a* (*n* = 3). Data are shown as the mean ± SEM. Statistics used included 1-way ANOVA. *P < 0.05, ***P < 0.001.

ATP7A/7B is a well-established intracellular copper transporter responsible for delivering copper to the Golgi apparatus (16, 17). To investigate whether ATP7A/7B participates in the copper-dependent activation of LOX within cells, immunohistochemical analyses was conducted. We found ATP7A, but not ATP7B, was notably enhanced in human kidney tissues diagnosed with fibrosis when compared with those without fibrosis (Figure 4A and Supplemental Figure 5A). In mice, IR-induced renal fibrosis was also associated with upregulated ATP7A, but not ATP7B, particularly in tubular epithelial cells (Figure 4, B and C; Supplemental Figure 5, B and C; and Supplemental Figure 6). To determine the specific role of ATP7A in renal fibrosis, we generated renal-specific *Atp7a* knockdown mice as described in the Methods section (detailed information about AAV injection can be found in Supplemental Figure 7). We found a significant decrease in copper levels within the Golgi apparatus after knockdown of *Atp7a* (Figure 4D). Additionally, treating NRK-52E cells with TGF-β1 resulted in an increase in ATP7A expression, while ATP7B remained unchanged (Figure 4E and Supplemental Figure 5D). Immunofluorescence staining confirmed that ATP7A expression colocalized with Golgin-97 (the Golgi marker) in NRK-52E cells, and ATP7A expression was increased following TGF-β1 treatment (Figure 4F). Next, we knocked down *Atp7a* in NRK-52E cells and observed a significant reduction in copper levels of Golgi apparatus induced by TGF-β1 (Figure 4G). These results suggest that ATP7A, rather than ATP7B, mediates the increase in Golgi copper level in renal fibrosis.

**Figure 5:**
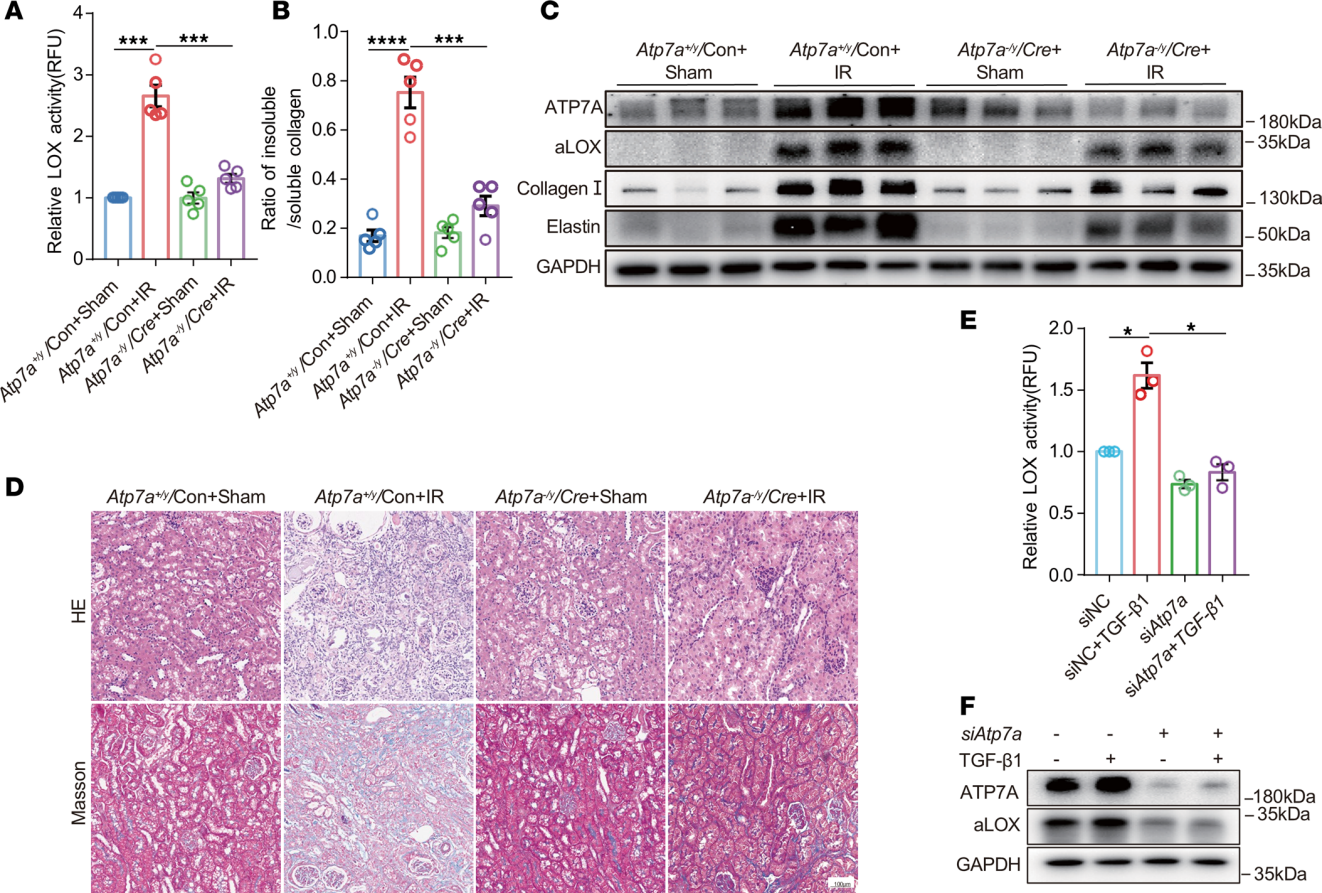
Downregulation of ATP7A in renal fibrosis reduces copper-dependent LOX activity. *Atp7a*^fl/y^ mice injected with AAV-Con or AAV-*Cre* were divided into the following 4 groups (*n* = 5/each group): *Atp7a^+/y^/*Con mice after sham operation; *Atp7a^+/y^/*Con mice after IR treatment; *Atp7a*^fl/y^/*Cre* mice after sham operation; and *Atp7a*^fl/y^/*Cre* mice after IR treatment (**A**–**D**). (**A**) LOX activity in kidney tissues was determined using the Fluorometric Lysyl Oxidase Assay Kit. (**B**) The ratio of insoluble/soluble collagen was analyzed in kidneys collected from IR-induced mice. (**C**) Western immunoblots analysis ATP7A, aLOX, collagen I, and elastin expression in mouse kidneys among different groups. (**D**) Representative images of H&E and Masson’s trichrome staining of kidney sections. Original magnification, ×200. Scale bar: 100 μm. (**E**) LOX activity was determined in the culture medium of NRK-52E cells among different groups (*n* = 3). (**F**) Western immunoblots of ATP7A and aLOX in NRK-52E cells among different groups (*n* = 3). Data are shown as the mean ± SEM. Statistics used included 1-way ANOVA. *P < 0.05, ***P < 0.001, ****P < 0.0001.

Then, we intended to investigate the role of ATP7A in the regulation of LOX activity. Downregulation of *Atp7a* in kidneys resulted in decreased LOX activity, reduced expression of aLOX, and a lower ratio of insoluble collagen to soluble collagen in IR-induced mice (Figure 5, A–C, and Supplemental Figure 5E). H&E staining and Masson’s trichrome staining revealed that the degree of tubular atrophy and dilatation and collagen deposition induced by IR was markedly ameliorated in *Atp7a^fl/y^* mice carrying AAV-*Cre* (Figure 5D and Supplemental Figure 5F). Downregulation of *Atp7a* in the *Atp7a^fl/y^/Cre* mice also reduced protein levels of fibrotic proteins, including collagen I and elastin (Figure 5C and Supplemental Figure 5E). In vitro, the downregulation of *Atp7a* in NRK-52E cells significantly reduced the TGF-β1–induced increase in LOX activity (Figure 5, E and F, and Supplemental Figure 5G). These findings indicate that Golgi ATP7A probably regulates LOX activity by transferring copper to LOX.

### FBLN4 serves as a critical mediator in the formation of ATP7A-LOX complex and modulates LOX activity.

**Figure 6:**
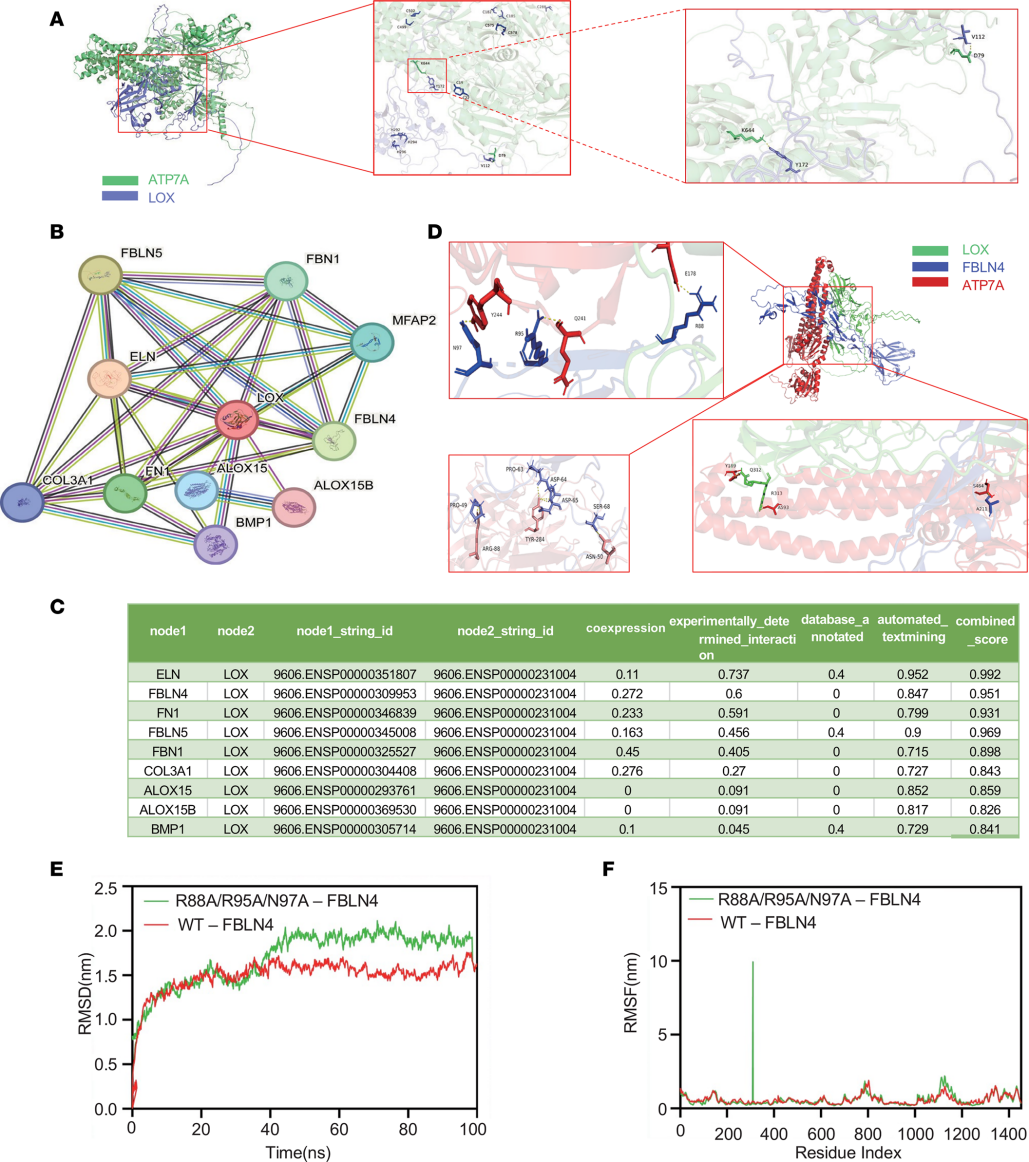
FBLN4 as a critical mediator of the ATP7A-LOX interaction by computational modeling analysis. (**A**) Molecular docking analysis of ATP7A and LOX. (**B**) The protein relationship between LOX and other proteins was forecasted using STRING tools (https://cn.string-db.org/). (**C**) The coexpression, experimentally determined interaction, database annotated, automated textmining, and combined score of aLOX and other molecules were calculated by STRING tools. (**D**) Molecular docking analysis of ATP7A, FBLN4, and LOX. (**E**) Molecular dynamics simulation stability analysis of the predicted ATP7A-FBLN4-LOX complex. Time-dependent root mean square deviation (RMSD) of backbone atoms for WT-FBLN4 (red curve) and the R88A/R95A/N97A-FBLN4 mutant (green curve) within the ternary complex. The trajectories reflect the overall conformational stability relative to the initial structure over a 100 ns simulation period. (**F**) Analysis of local residue flexibility within the predicted ATP7A-FBLN4-LOX complex. Per-residue root mean square fluctuation (RMSF) of backbone atoms for WT-FBLN4 (red curve) and the R88A/R95A/N97A-FBLN4 mutant (green curve).

Docking analysis revealed that residues K644 and D79 in ATP7A can form hydrogen bonds with the residues Y172 and V112 in LOX, respectively (Figure 6A). The affinity is judged by binding energy, –151.3 kcal/mol, indicating relatively weak affinity. Based on this, it is speculated that there may be other factors involved. According to the protein-protein interaction (PPI) network analysis we found that FBLN4 showed the highest experimentally determined interaction score (Figure 6, B and C). Unexpected, in the presence of FBLN4, docking analysis revealed that the binding energy was markedly increased from the original –151.3 kcal/mol to –309.0 kcal/mol between ATP7A and LOX, with a larger absolute value indicating stronger binding (Figure 6D).

To determine whether FBLN4 is essential for the ATP7A-LOX interaction, we generated mutant and WT FBLN4 proteins using GROMACS 2019.5, with the mutant variant containing alanine (A) substitutions at the R88, R95, and N97 sites. Molecular dynamics simulation demonstrated that the FBLN4 mutant reduced the stability of the ATP7A-LOX-FBLN4 complex compared with WT FBLN4, as evidenced by decreased root mean square deviation (RMSD) and root mean square fluctuation (Figure 6, E and F) (18).

**Figure 7:**
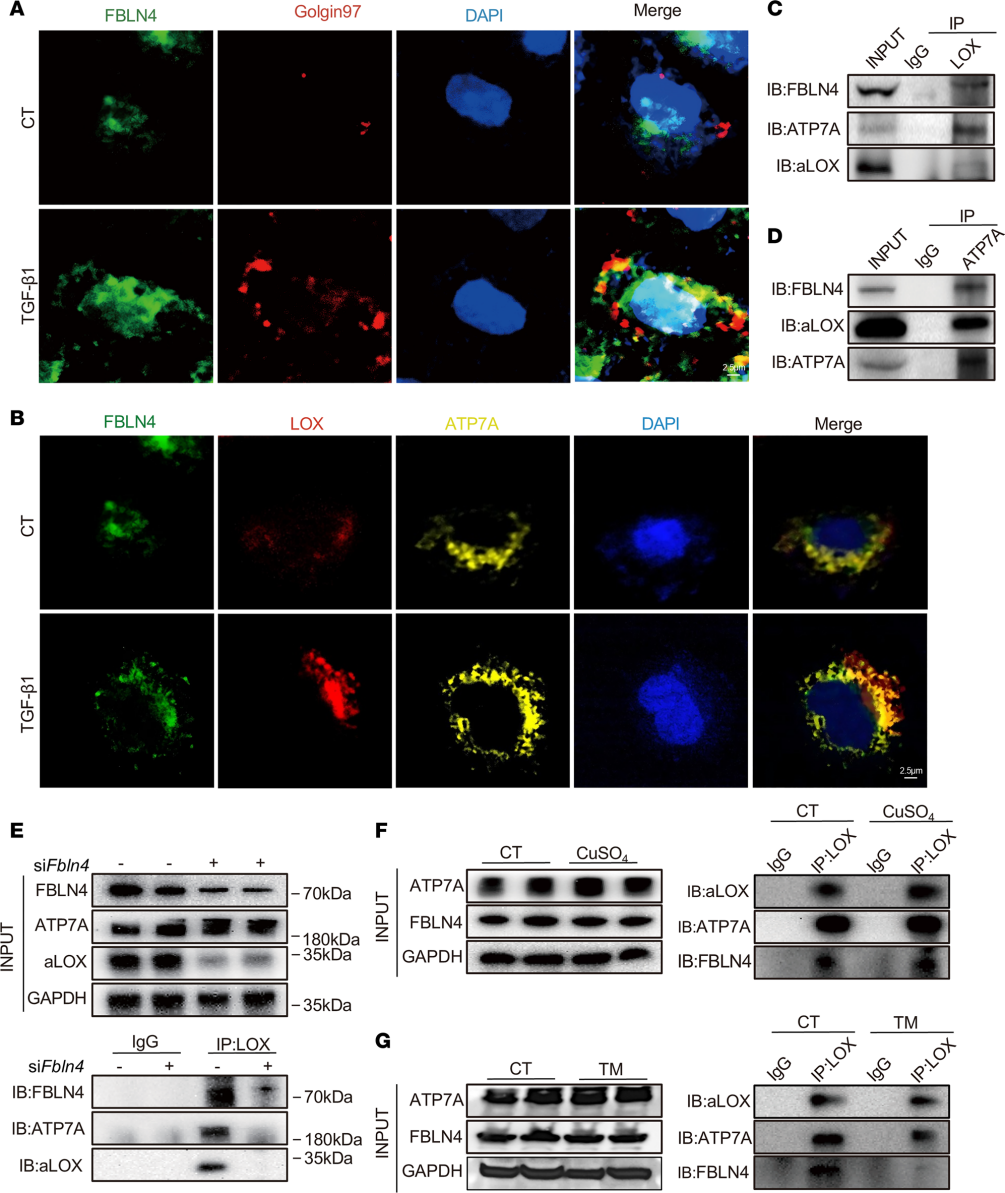
FBLN4 is essential for the formation of the ATP7A-LOX complex in renal fibrosis. (**A**) The colocalization of FBLN4 (green) with the Golgi apparatus (red) was analyzed by immunofluorescent costaining in NRK-52E cells without (CT) or with TGF-β1 treatment (*n* = 3). Original magnification, ×1,000. Scale bar: 2.5 μm. (**B**) The colocalization of FBLN4 (green) with ATP7A (yellow) and LOX (red) was analyzed by immunofluorescent costaining in NRK-52E cells without (CT) or with TGF-β1 treatment (*n* = 3). Original magnification, ×1,000. Scale bar: 2.5 μm. (**C** and **D**) Coimmunoprecipitation showing the interaction among ATP7A, FBLN4, and aLOX proteins in NRK-52 cells. (**E**) Coimmunoprecipitation showing the interaction among ATP7A, FBLN4, and aLOX after downregulating *Fbln4*. (**F** and **G**) Coimmunoprecipitation showing the interaction among ATP7A, FBLN4, and aLOX proteins in NRK-52 cells under CuSO_4_- and TM-treated conditions. INPUT indicates total protein. IP indicates the antibody used to pull down the interacting proteins. IgG represents the negative control.

Furthermore, we found a colocalization of FBLN4 and Goligin-97, the Golgi marker, in NRK-52E cells with TGF-β1 treatment (Figure 7A). Immunofluorescent assay revealed a marked increase in colocalization areas among FBLN4, ATP7A, and LOX upon TGF-β1 stimulation (Figure 7B). To demonstrate the physical interaction among ATP7A, FBLN4, and LOX, immunoprecipitation assay clearly showed that either ATP7A or FBLN4 could bind to LOX in NRK-52E cells (Figure 7C). Immunoprecipitation of ATP7A also confirmed the interaction among ATP7A, FBLN4, and LOX (Figure 7D), suggesting that these proteins may form a complex. Additionally, knockdown of *Fbln4* abolished the interaction between LOX and ATP7A (Figure 7E).

To further investigate the regulatory mechanism of ATP7A-LOX complex stability, we explored the role of copper, a critical cofactor for LOX activity. Coimmunoprecipitation assays revealed that CuSO_4_ treatment enhanced the interaction between ATP7A and LOX, whereas copper chelation TM reduced their binding in a dose-dependent manner (Figure 7, F and G). These results uncovered the existence of a copper-dependent FBLN4/ATP7A/LOX pathway for promoting LOX activity.

### Inhibition of FBLN4 disrupts copper transfer from ATP7A to LOX, thereby suppressing renal fibrosis.

**Figure 8:**
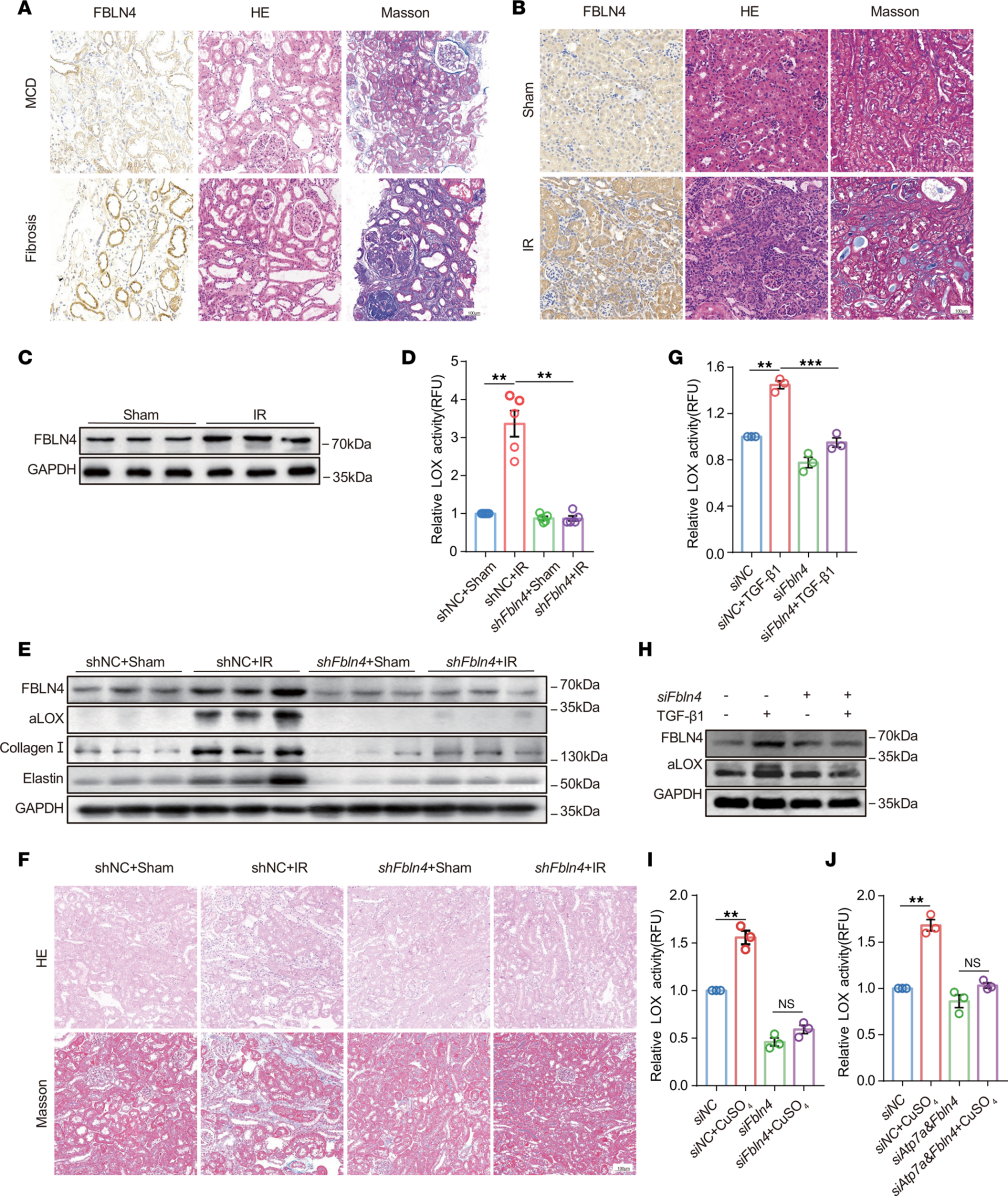
Disruption of FBLN4-mediated copper transfer from ATP7A to LOX suppresses renal fibrosis. (**A**) Representative images of immunohistochemical staining with FBLN4, H&E, and Masson’s trichrome in kidney sections from patients with and without renal fibrosis (*n* = 5). Original magnification, ×200. Scale bar: 100 μm. (**B**) Representative images of immunohistochemical staining with FBLN4, H&E, and Masson’s trichrome in kidney subjected to IR injury (*n* = 5). Original magnification, ×200. Scale bar: 100 μm. (**C**) Western immunoblots of FBLN4 in kidney tissues among different groups (*n* = 5). All mice were randomly divided into 4 groups (*n* = 5/each group): sham-operated mice treated with AAV9-Con (shNC + sham), sham-operated mice treated with AAV9-sh*FBLN4* (sh*FBLN4* + sham), IR mice treated with AAV9-Con (shNC + IR), and IR mice treated with AAV9- sh*FB LN4* (sh*FBLN4* + IR) (**D**–**F**). (**D**) LOX activity in kidney tissues was determined using the Fluorometric Lysyl Oxidase Assay Kit. (**E**) Western immunoblots analysis of FBLN4, aLOX, collagen I, and elastin expression in mouse kidneys among different groups. (**F**) Representative images of H&E and Masson’s trichrome staining of kidney sections. Original magnification, ×200. Scale bar: 100 μm. (**G**) LOX activity as determined using the Fluorometric Lysyl Oxidase Assay Kit in the culture medium of NRK-52E cells after *Fbln4* knockdown (*n* = 3). (**H**) Western immunoblots of FBLN4 and aLOX in NRK-52E cells among different groups (*n* = 3). (**I** and **J**) LOX activity was determined in the culture medium of NRK-52E cells treated with CuSO_4_ after downregulation of *Fbln4* alone and downregulation of both *Atp7a* and *Fbln4* (*n* = 3). Data are shown as the mean ± SEM. Statistics used included 1-way ANOVA. **P < 0.01, ***P < 0.001.

In this study, we clarify whether FBLN4 participates in the regulation of LOX activity by disrupting copper transfer in renal fibrosis. We observed an increased expression of FBLN4 in human kidney tissues diagnosed with fibrosis when compared with those with MCD (Figure 8A and Supplemental Figure 8A). In mice, IR-induced renal fibrosis was also associated with upregulated FBLN4 (Figure 8, B and C, and Supplemental Figure 8, B and C). To explore the role of FBLN4 in modulation of LOX activity, we depleted *Fbln4* by delivering AAV9-sh *Fbln4* into mice. AAV9-sh *Fbln4* depleted FBLN4 efficiently in kidney tissues (Figure 8E and Supplemental Figure 8D). A reduction in renal LOX activity and aLOX expression in fibrotic kidney tissues were when *Fbln4* was downregulated (Figure 8, D and E, and Supplemental Figure 8D). Consistently, downregulation of *Fbln4* markedly exacerbated fibrotic protein deposition induced by IR injury (Figure 8, E and F, and Supplemental Figure 8, D and E). In vitro, the downregulation of *Fbln4* in NRK-52E cells significantly reduced the TGF-β1–induced increase in LOX activity (Figure 8, G and H, and Supplemental Figure 8F). Knockdown of ATP7A with shRNA resulted in an upregulation of LOX activity, whereas double knockdown of ATP7A and FBLN4 failed to rescue LOX activity in CuSO4-treated NRK-52E cells (Figure 8, I and J). Interestingly, downregulation of *Fbln4* did not significantly alter the mRNA expression level of LOX (Supplemental Figure 9), suggesting that FBLN4 regulates LOX activity through posttranslational modification rather than transcriptional regulation. Together, these results suggest that FBLN4 facilitates the transfer of copper from ATP7A to LOX, thereby promoting LOX activation and contributing to renal fibrosis.

## Discussion

**Figure 9:**
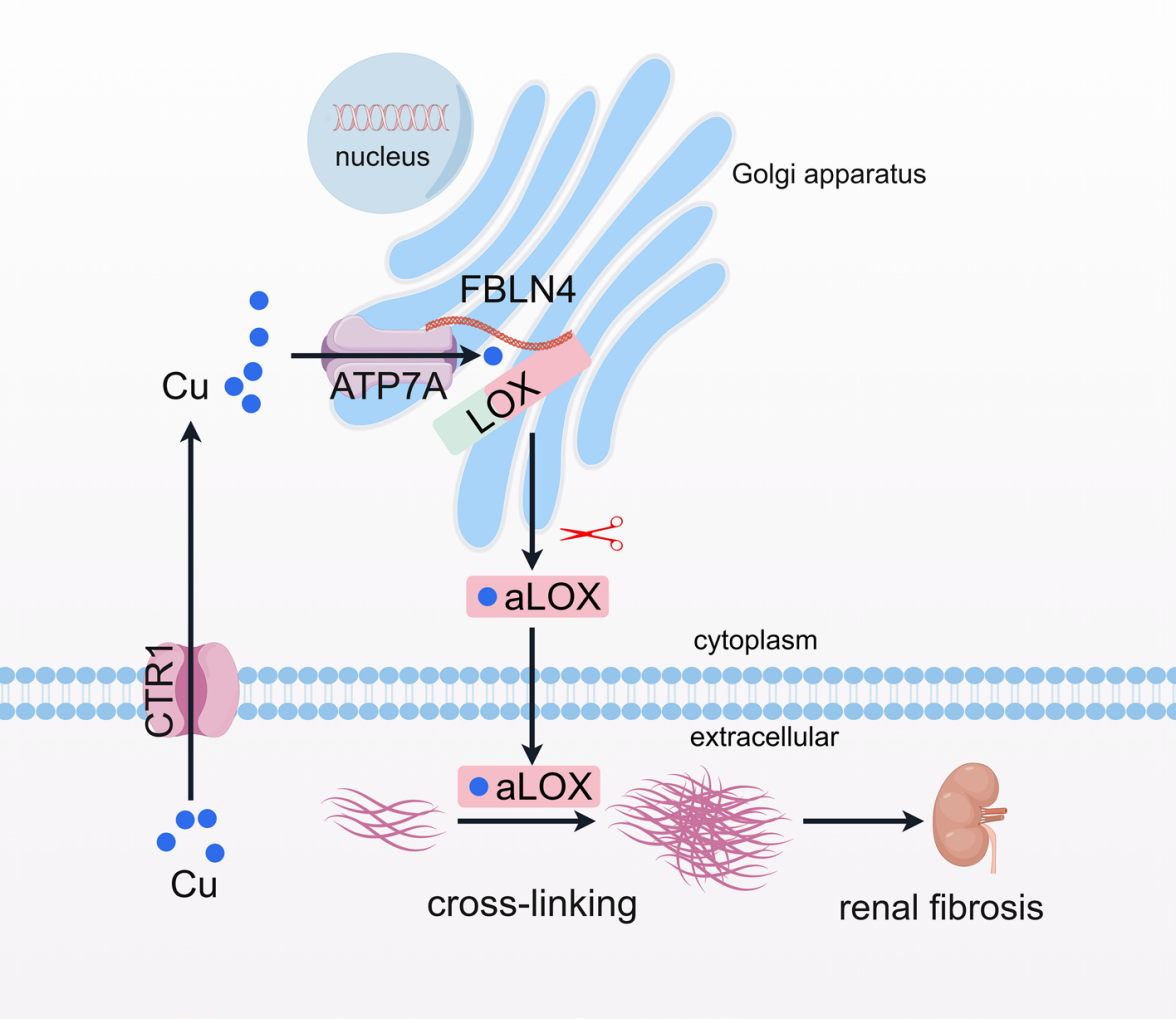
Diagram depicting the regulatory mechanism of LOX activity in renal fibrosis. The study illuminates the connection between LOX activity and copper in renal fibrosis, highlighting the mediation of LOX activity by copper through the ATP7A-FBLN4 complex (created using Figdraw; https://www.figdraw.com/).

This study revealed an association of intra-Golgi apparatus copper accumulation with LOX activity–dependent ECM cross-linking and renal fibrosis. We showed that, in addition to LOX expression, LOX activity was increased during the process of renal fibrosis, accompanying an increase of intracellular copper levels, particularly within the Golgi apparatus. We further demonstrated that ATP7A regulates copper-dependent LOX activity through the induction of intra-Golgi copper accumulation and that this process was involved in copper-dependent FBLN4-mediated the interaction of ATP7A with LOX (Figure 9). This study demonstrates that the induction of copper accumulation within the Golgi apparatus, resulting in a reduction increase of LOX activity, contributes to reduced ECM degradation and enhanced renal fibrosis. Both reducing intracellular Golgi copper levels and targeting the inhibiting of copper transport in the Golgi apparatus can block this process.

Our previous studies show that upregulation of LOX expression contributes to ECM cross-linking and the progression of renal fibrosis in IR-induced mouse injury (5). However, the ECM serves as a dynamic structural and regulatory scaffold that is essential for cell fate determination, differentiation, and intercellular communication during development, tissue homeostasis, and repair (10, 11). Increasing studies reported that LOX activity, rather than LOX expression, plays a more critical role in fibrotic diseases, including inflammatory diseases, cancer metastasis, and progression (19–21). Cu-dependent LOX activity is considered as the primary role of the assembly of ECM. Among these, the main organelle in which LOX absorbs copper is the Golgi apparatus (12, 22). To our knowledge, the role of Cu-dependent LOX activity in renal fibrosis has not yet been reported. In the current study, our findings demonstrated that the copper level within the Golgi apparatus increases in a fibrotic environment. Both reduction of intracellular copper and depletion of Golgi copper inhibit LOX activity and ameliorate renal fibrosis, highlighting the essential role of Golgi-resident copper in LOX activation.

ATP7A and ATP7B are copper-chaperone proteins responsible for mediating the translocation of intracellular copper ions to the Golgi apparatus (11, 17, 22). Gene defect or mutation encoding ATP7A in intestinal epithelial cells on the X chromosome results in congenital copper metabolism disorders known as Menkes syndrome (13, 23). Hepatolenticular degeneration (Wilson’s disease) is an autosomal recessive genetic disorder caused by mutations in the ATP7B gene, leading to excessive copper accumulation in hepatocytes (24, 25). The loss of ATP7B function directly drives liver fibrosis through disrupted copper metabolism (26, 27). In our study, we demonstrated increased ATP7A, but not ATP7B, expression in renal fibrosis, with ATP7A upregulation induced by TGF-β1. ATP7A is the primary responder to transport copper to Golgi apparatus, which subsequently upregulates LOX activity and promotes renal fibrosis. This study suggests that both ATP7A and ATP7B contribute to organ fibrosis progression via distinct tissue-specific mechanisms.

Another important issue is how ATP7A transfers copper ions to LOX. To address this question, we assessed the binding affinity between ATP7A and LOX. Unfortunately, the binding activity between ATP7A and LOX was weak, which led us to hypothesize that there might be other accessory proteins involved. Based on protein interaction network analysis, we found that FBLN4 plays a pivotal role in the process of the copper transfer from ATP7A to LOX. Patients harboring mutations in the *Fbln4* gene manifest clinical symptoms such as vascular malformations, aneurysms, and skin laxity (28–30). Similarly, *Fbln4-*deficient mice exhibit aortic aneurysms and emphysema, with notable damage to elastic fibers and abnormal collagen fibers (31). These pathological manifestations resemble the phenotype observed in *Lox*-deficient mice (32–34), suggesting a connection between FBLN4 and LOX. Researchers previously observed a decrease in LOX activity in connective tissues, including blood vessels, bones, and skin, in mice lacking FBLN4 (35). However, the role of FBLN4 in the regulation of LOX in renal fibrosis was unknown. In the presence of FBLN4, docking analysis revealed that the binding energy was markedly increased between ATP7A (Y970, A1374) and LOX (Q312, R313) (Figure 6D). In our study, we observed an elevated binding energy from –151.3 kcal/mol to –309.0 kcal/mol between ATP7A and LOX in the presence of FBLN4. To further elucidate these intricate interactions, coimmunoprecipitation and multi-immunofluorescent assays were employed to confirm the association among ATP7A, FBLN4, and LOX. Notably, both in vivo and in vitro downregulation of *Fbln4* led to a marked decrease in LOX enzymatic activity. Intriguingly, this reduction in LOX activity following *Fbln4* depletion was not reversed by copper supplementation, suggesting an essential role for FBLN4 in copper-dependent LOX activation. In contrast, partial restoration of LOX activity was observed after *Atp7a* knockdown followed by copper treatment. Further coimmunoprecipitation experiments demonstrated that *Fbln4* knockdown significantly diminished the interaction between LOX and ATP7A.

There are still several limitations in this study, including (a) the lack of an effective method for specific conditional chelation of copper within the Golgi apparatus and (b) the requirement of a large sample size for LOX activity detection, which precludes validation in clinical spices. Our future research will focus on achieving precise, specific regulation of Golgi copper ions.

In conclusion, this study provides evidence that FBLN4-ATP7A delivers copper to the Golgi apparatus, where it interacts with and transfers copper to LOX, leading to LOX activation and, ultimately, promoting renal fibrosis. Our current study focuses on the role of LOX activity in renal fibrosis, and our findings provide insight and aim to provide a comprehensive elucidation of the regulatory mechanisms underlying LOX regulation from transcriptional expression to functional activation. Our findings suggest that inhibiting copper accumulation in the Golgi may represent a precise therapy for LOX-driven renal fibrosis.

## Methods

### Sex as a biological variable.

In this study, only male mice were utilized to minimize variability associated with hormonal cycles and to ensure consistency across experimental groups. Consequently, the potential for sex-specific differences could not be evaluated.

### Human renal biopsies sections.

Renal biopsy sections from humans were obtained as part of routine clinical diagnostic investigations and were selected based on the pathological diagnosis of fibrosis score. These sections were utilized for immunohistochemical staining. The investigations adhered to the principles outlined in the Declaration of Helsinki, and the ethical approval was granted by the Research Ethics Committee of Tongji Hospital. Informed consent was obtained from the patients prior to their participation in the study (NO. K-W-2021-012).

### Animal models and treatments.

*Ctr1^fl/fl^* mice harboring loxP sites flanking exon 2 and exon 3 of Slc31a1-201 (ENSMUST00000084526.11) were generated by Jiangsu Gempharmatech Co. Ltd. *Ggt1-Cre* mice were purchased from Cyagen Biosciences Inc. *Ctr1^fl/fl^* mice were crossed with *Ggt1-Cre* mice on a C57BL/6J background to generate *Ctr1^fl/fl^/Ggt1-Cre^–^* and *Ctr1^fl/fl^/Ggt1-Cre^+^* mice. For the analysis of *Ctr1*-manipulated mice, male *Ctr1*^fl/fl^/*Cre*^–^ and *Ctr1*^fl/fl^/*Cre*^+^ mice were randomly divided into 4 groups: *Ctr1*^fl/fl^/*Cre*^–^ mice + sham; *Ctr1*^fl/fl^/*Cre*^–^ mice + IR; *Ctr1*^fl/fl^/*Cre*^+^ mice + sham; and *Ctr1*^fl/fl^/*Cre*^+^ mice + IR.

*Atp7a^fl/y^* mice on a C57BL/6J background harboring loxP sites flanking exon 3 and exon 5 of *Atp7a-201* (ENSMUST00000055941.6) were generated by Jiangsu Gempharmatech Co. Ltd. Both systemic deletion of ATP7A and conditional deletion using a floxed allele with Ggt1-Cre result in embryonic lethality in mice (36). Male *Atp7A^fl/y^* mice were divided into 2 groups that received subcapsular injections of adeno-associated virus vectors (AAV, Serotype 9, HanBio) carrying either the Cre recombinase (AAV-*Cre*) or (AAV-*Con*) respectively to generate Cre-mediated *Atp7a* knockdown (*Atp7a*^–/y^/*Cre*) and control mice (*Atp7a^+/y^*/Con). For the analysis of *Atp7a-*manipulated mice, male mice were randomly divided into 4 groups: *Atp7a^+/y^*/Con mice + sham; *Atp7a^+/y^*/Con mice + IR; *Atp7a*^–/y^/*Cre* mice + sham; and *Atp7a*^–/y^/*Cre* mice + IR.

shRNA targeting mouse *Fbln4* gene (sequence: 5’-GGGACTTCTACATTAGGCAAATCAA-3’) was designed and oligonucleotides were synthesized by HanBio. The AAV9 vector harboring shRNA targeting the *Fbln4* gene (AAV9-sh*Fbln4*) was constructed by HanBio. An empty AAV9 vector (AAV9-Con) was used as the control. All mice were randomly divided into 4 groups: sham-operated mice treated with AAV9-Con (shNC + sham), sham-operated mice treated with AAV9-sh*Fbln4* (sh*Fbln4* + sham), IR mice treated with AAV9-Con (shNC + IR), and IR mice treated with AAV9-sh*F*bln4(sh*Fbln4* + IR). The viruses were delivered by subcapsular injections, and the animals were sacrificed 28 days after surgery for sample harvesting.

Male C57BL/6 mice were purchased from the Silaike Laboratory. All animal studies were conducted in male mice aged 6–8 weeks at the time of surgery. Mice were subjected to IR surgery, FA treatment, and UUO surgery as described previously (5). After undergoing IR or UUO operation, mice received the irreversible LOX activity inhibitor BAPN (100 mg/kg/d, A3134, Sigma Aldrich) or vehicle (normal saline) continuously for 28 days by gavage. Mice were administered TM, which chelates copper both intracellularly and extracellularly, at a dose of 30 mg/kg body weight per day via intragastric gavage (323446, Sigma-Aldrich) for 28 days following IR injury, while the vehicle controls received an equivalent volume of DMSO (37). Mice were euthanized with 1% pentobarbital sodium (0.009 ml/g, Sigma-Aldrich) through intraperitoneal injection.

### Histopathology.

For interstitial fibrosis, mouse kidney tissues were embedded in paraffin to prepare sections (4 μm). The sections were subjected to H&E (Solarbio, G1120) and Masson’s trichrome (KeyGEN BioTECH, KGE1113-8) staining. Images were analyzed using a Nikon Eclipse DS-Ri2 microscope. H&E slides were scored on a scale from 0 to 4: 0, normal; 1, mild (<25% of the cortex); 2, moderate (25%–50%); 3, severe (50%–75%); 4, extensive damage (>75%) (38). The scoring and the positive area of collagen fibers (Masson’s trichrome, blue) was calculated in 10 bright-field images (×200) in the eyepiece of the microscope (39).

### Immunohistochemistry.

The sections were incubated with primary antibodies against LOX (Invitrogen, MA5-35123, 1:50), ATP7A (Affinity Biosciences, DF8506, 1:100), ATP7B (Abcam, ab131208, 1:50), FBLN4 (Affinity Biosciences, AF5209, 1:50) and CTR1 (Abmart, T510261, 1:200). Images were analyzed using a Nikon Eclipse DS-Ri2 microscope. Quantitative evaluation involved the use of Image-Pro Plus 6.0 (Media Cybernetics, Rockville, MD, USA) (40). Positive signals and the number of positive cells in the kidney were quantified in 10 bright-field images (×200) in the eyepiece of the microscope.

### Western blotting.

Proteins were extracted from kidney tissues or NRK-52E cells, separated using sodium dodecyl sulfate-polyacrylamide gel electrophoresis gels, and subsequently transferred onto polyvinylidene difluoride membranes (Millipore). Antibodies against LOX (Invitrogen, MA5-35123, 1:1,000), ATP7A (Affinity Biosciences, DF8506, 1:1,000), ATP7B (Santa Cruz Biotechnology, sc-373964, 1:1,000), FBLN4 (Affinity Biosciences, AF5209, 1:1,000), Collagen I (Abcam, ab260043, 1:2,000), Elastin (Santa Cruz Biotechnology, sc-58756, 1:2,000), CTR1 (Abmart, T510261S, 1:2,000) and GAPDH (Affinity Biosciences, AF7021, 1:5,000) were used for Western blotting.

### Real-time qPCR.

Total RNA was extracted from tissues and cells using TRIzol (Invitrogen, 15596026) followed by chloroform extraction according to the manufacturer’s protocol. RNA reverse transcription was performed using the PrimeScript RT Reagent Kit (Takara, RR036A). Real-time quantitative PCR (qPCR) was performed with Applied Biosystems QuantStudio 5 (Thermo Fisher Scientific). The relative expression of each gene was determined by normalizing to GAPDH. The primers used in this study target the following genes: mouse-derived *Gapdh*, *Atp7a*, and *Atp7b*; as well as rat-derived *Gapdh*, *Fbln4*, and *Lox* (Supplemental Table 1).

### Cell culture and treatment.

NRK-52E cells were purchased from FuHeng Biology and cultured in DMEM (FuHeng Biology) supplemented with 10% FBS, 100 U/mL penicillin, and 100 μg/mL streptomycin (FuHeng Biology) at 37°C in a humidified atmosphere of 5% CO_2_ and 95% air. NRK-52E cells were treated with TGF-β1 (10 ng/mL, R&D Systems), TM (1.5 μM, 323446, Sigma-Aldrich), and CuSO_4_ (25 μM, 209198, Sigma-Aldrich) either individually or in combination for 48 hours. Knockdown of *Atp7a* and *Fbln4* was performed by infection with respective siRNA. The targeting sequence of si*Atp7a* is 5′-GGACAGAGGAGUCCUUCAGAATT-3′. The targeting sequence of si*Fbln4* is 5′-GCUCUUGUGUGGAUGUGAATT-3′.

### Transfection of shRNA.

shRNA targeting rat CTR1 gene (sequence: 5′-GACCTACAATGGGTACCTA-3′) was designed and oligonucleotides were synthesized by HarO Bio. The construction of CTR1 shRNA-pLVshRNA-mCherry (2A) puro vector and a lentiviral vector were described by previous study (5). The virus was harvested and transferred into NRK-52E cells. Puromycin was used to screen for cells that were successfully transfected with the lentivirus.

### Immunofluorescence staining.

Primary antibodies used were rabbit anti-ATP7A (1:200; Affinity Biosciences, DF8506), mouse anti-Golgin-97 (1:25, Santa Cruz, sc-59820), rabbit anti-FBLN4 (1:50, Affinity Biosciences, AF5209), rabbit anti-LOX (1:50, Invitrogen, MA5-35123), rabbit anti-CTR1 (1:100, Genuin Biotechnologies, 1823), and rabbit anti-AQP1 (1:50, Proteintech, 20333-1-AP). Secondary antibodies were incubated in blocking buffer for 1 hour at room temperature. Samples were extensively washed with 0.1 M PBS. When primary antibodies were derived from different species for immunofluorescence co-staining, signal was visualized with fluorophore-conjugated goat secondary antibodies (1:200; goat anti-rabbit-488, A-11008, Invitrogen; goat anti-mouse-568, A-11004, Invitrogen).When primary antibodies were from the same species, the 4-Color IHC Kit (Absin, abs50028) was used for fluorescent labeling.

### Isolation of Golgi apparatus.

Golgi apparatuses from cells were isolated using a commercial Minute Golgi Apparatus Enrichment Kit (Invent, GO-037) according to the manufacturer’s protocol. The protein concentration of the isolated Golgi apparatuses was assessed using the Bicinchoninic Acid Assay (Beyotime, P0010).

### LOX activity.

LOX activity refers to its catalytic function, which was assessed in this study using 2 methods: measurement of enzymatic activity via Fluorometric Lysyl Oxidase Assay Kit (AAT Bioquest, 15255) (6) and detection of the active LOX fragment by Western blotting (Invitrogen, MA5-35123, 1:1,000, 32 kDa band represents the fragment of the LOX activity) (10).

### Collagen cross-linking.

Kidney tissues were weighted after harvested and snap-frozen in liquid nitrogen. Homogenized kidney tissues were then treated with 0.5 mol/L acetic acids (695092, Sigma-Aldrich) overnight and then digested with 5 mg/mL pepsin overnight (10718500, Sigma-Aldrich). The pepsin-soluble and pepsin-insoluble collagen fractions were separated by centrifugation at 14,000*g* for 10 minutes and then analyzed using a commercialized assay kit (Hydroxyproline Assay Kit, BC0250, Solarbio).

### Inductively coupled plasma mass spectrometry.

Samples were treated with concentrated nitric acid and subsequently heated to a temperature of 100°C. This process was continued until the solution reached a state of complete clarity and was free from any precipitate. Following this, the sample underwent dilution with double-distilled water to reduce the concentration of the nitric acid to below 5%. Standard samples for detecting copper ions were purchased (Sero, 1103129) and used for quantification. The parameter settings for copper ion detection in the sample using inductively coupled plasma mass spectrometry (ICP-MS) (Fudan University) are as previously described (6).

### Molecular docking.

The 3D structures of Fibulin-4 (Uniprot ID O95967), ATP7A (Uniprot ID Q04656), and LOX (Uniprot ID P28300) were determined by AlphaFold. Then, the molecular simulation module of HEX (https://hex.loria.fr/) was used to predict protein interactions (41, 42).

### Building of PPI network.

PPI network analysis was performed on target genes combined with FBLN4 using the STRING database (https://string-db.org/), and interactions with a confidence score ≥0.8 were selected to construct PPI network.

### Molecular dynamics simulations.

Molecular dynamics simulations were performed using GROMACS 2019.5 to elucidate conformational changes in WT and mutant FBLN4 complexes. Using the lowest-energy docking structures, systems were parameterized with AMBER ff14SB and GAFF force fields, solvated in explicit SPE/C water with a 10 Å margin, and neutralized to 0.15 M NaCl. Energy minimization employed steepest descent and conjugate gradient algorithms (5,000 steps each), followed by NVT (Langevin thermostat, 298 K), and NPT (Berendsen barostat, 1 atm) equilibration. Upon confirming system stability, 100 ns unrestrained production runs were conducted at 298 K on a GPU-accelerated server. Trajectory dynamics were analyzed using root mean square deviation (43).

### Statistics.

All data examined are expressed as the mean ± SEM. Two-tailed Student’s *t* test was used to compare differences between 2 groups. One-way ANOVA was performed to analyze differences among multiple groups. *P* < 0.05 were considered statistically significant. SPSS 21.0 statistical software was used for statistical analyses.

### Study approval.

Approval for all human studies was granted by the Research Ethics Committee of Tongji Hospital. Informed consent was obtained from the patients prior to their participation in the study (no. K-W-2021-012). The protocol for animal use was approved by the Ethics Committee of Hubei Animal Experiment Center of Tongji University and was performed in accordance with institutional guidelines (no. Tj-HB- LAC-2023-38).

### Data availability.

Values for all data points in graphs are reported in the Supporting Data Values file. Kidney single-cell expression data (Healthy Mouse Dataset, ref. 15, and RBK RID: 14-4KBC) were obtained from the Kidney Interactive Transcriptomics online analysis platform (http://humphreyslab.com/SingleCell/).

## Author contributions

CY and WZ designed the study. Both WZ and Y Zheng performed the experiments, analyzed, and interpreted the results. WZ and Y Zheng are co–first authors, listed in chronological order of their contributions: WZ initiated the foundational research, primary experiments, and wrote the manuscript; Y Zheng subsequently led the comprehensive revision, including all experimental validations, data reanalysis, figure revisions, and manuscript rewriting. Yuqing Liu, Yiguo Liu, and JL assisted with the main experiments. YN and YY provided essential reagents and techniques for this study and reviewed the manuscript. CY, Y Zhang, WZ, and XZ approved the final version of the manuscript. CY conceived and supervised the study.

## Conflict of interest

The authors have declared that no conflict of interest exists.

## Funding support

- The National Natural Science Foundation of China (U25A2032, no. 82470764, no. 82170696, no. 82300769).
- The Project of Shanghai Municipal Commission of Science and Technology (no. 23Y11908900).
- The scientific research project of Shanghai Municipal Health Commission (20194Y0115).

## Supplementary Material

Supplemental data

Unedited blot and gel images

Supporting data values

## Version 1 05/08/2026

Electronic publication
