# Supplementary material for: ATP7A-fibulin-4 complex delivers copper in the Golgi to activate LOX in renal fibrosis
Source: JCI Insight. 2026 May 8;11(9):e199028. doi: 10.1172/jci.insight.199028 (PMC13232011; doi:10.1172/jci.insight.199028)
Supplement: Supplemental data [file jciinsight-11-199028-s153.pdf]

SUPPLEMENTAL FIGURE LEGENDS

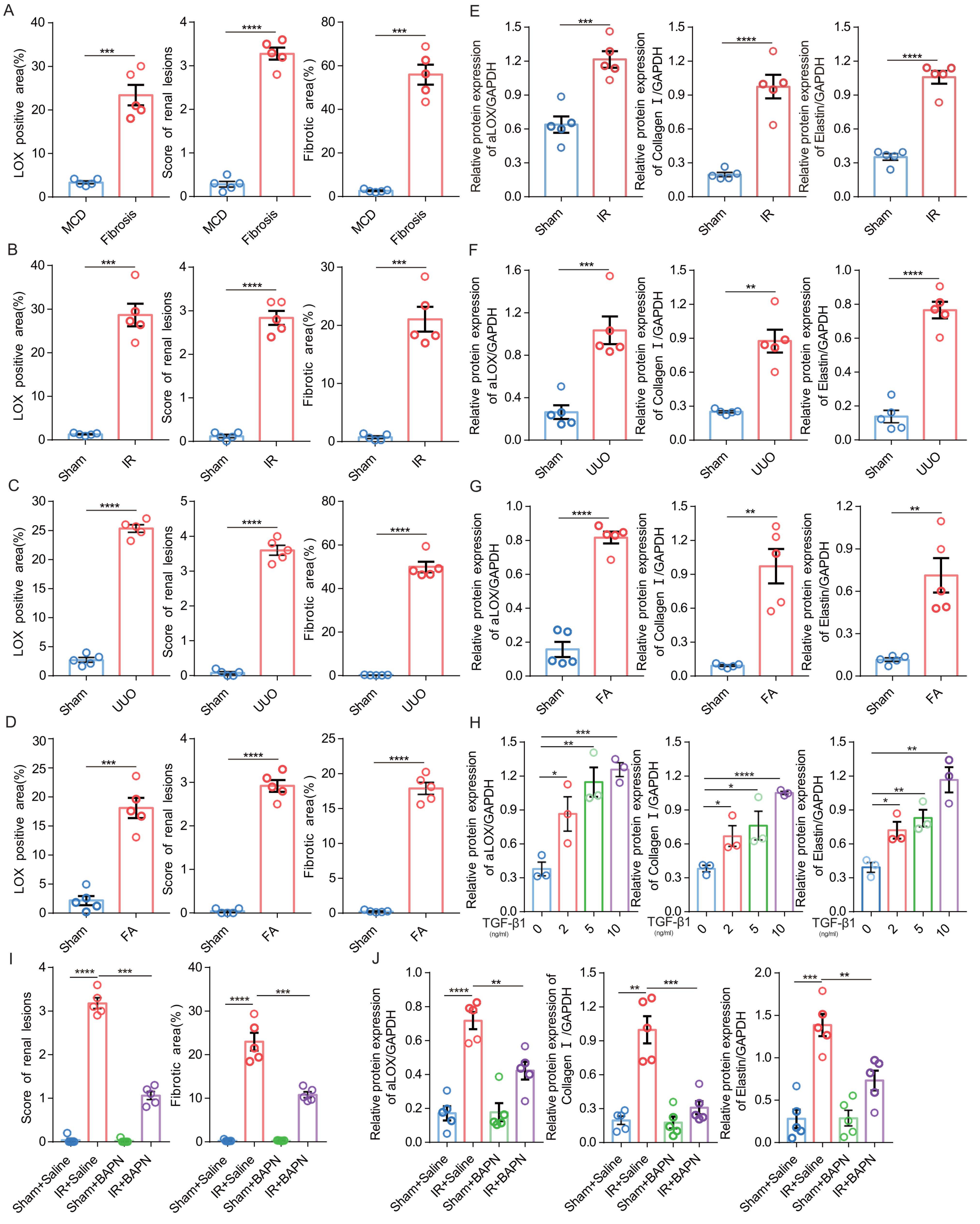

Supplemental Figure 1. The quantitative analysis data of Figure 1.

(**A**) Quantitative analysis of immunohistochemical staining with LOX antibody, HE and Masson's staining in kidney sections from patients with and without renal fibrosis (*n* = 5). Original magnification, ×200. Bar = 100 μm.

(**B-D**) Quantitative analysis of immunohistochemical staining of LOX, HE and Masson's staining in kidney sections from renal fibrosis models induced by IR, UUO, FA (*n* = 5). Original

magnification, ×200. Bar = 100 μm.

(**E-G**) Quantitative analysis of aLOX (activated aLOX), Collagen I and Elastin levels detected by Western immunoblots in kidneys collected from IR-, UUO- and FA- mice models (*n* = 5).

(**H**) Quantitative analysis of the expression of aLOX, Collagen I and Elastin levels by Western immunoblots in NRK-52E cells treated with TGF-β1 (*n* = 3).

For BAPN treatment, four groups (*n* = 5 / each group) of C57BL/6 mice were prepared: Sham + Saline; Sham + BAPN (100 mg/kg/day); IR + Saline; and IR + BAPN (I-J).

(**I**) Quantitative analysis of HE and Masson's staining of kidney sections. Original magnification, ×200. Bar = 100 μm.

(**J**) Quantitative analysis of aLOX, Collagen I and Elastin levels in mouse kidneys detected by Western immunoblots.

Each bar represents the mean ± SEM, statistics used included a 2-tailed t test (2 groups, in A-G) or 1-way ANOVA (multiple groups, in H-J). *, $P < 0.05$, **, $P < 0.01$, ***, $P < 0.001$, ****, $P < 0.0001$. IR, ischemia-reperfusion injury; FA, folic acid-treated; UUO, unilateral urethral obstruction.

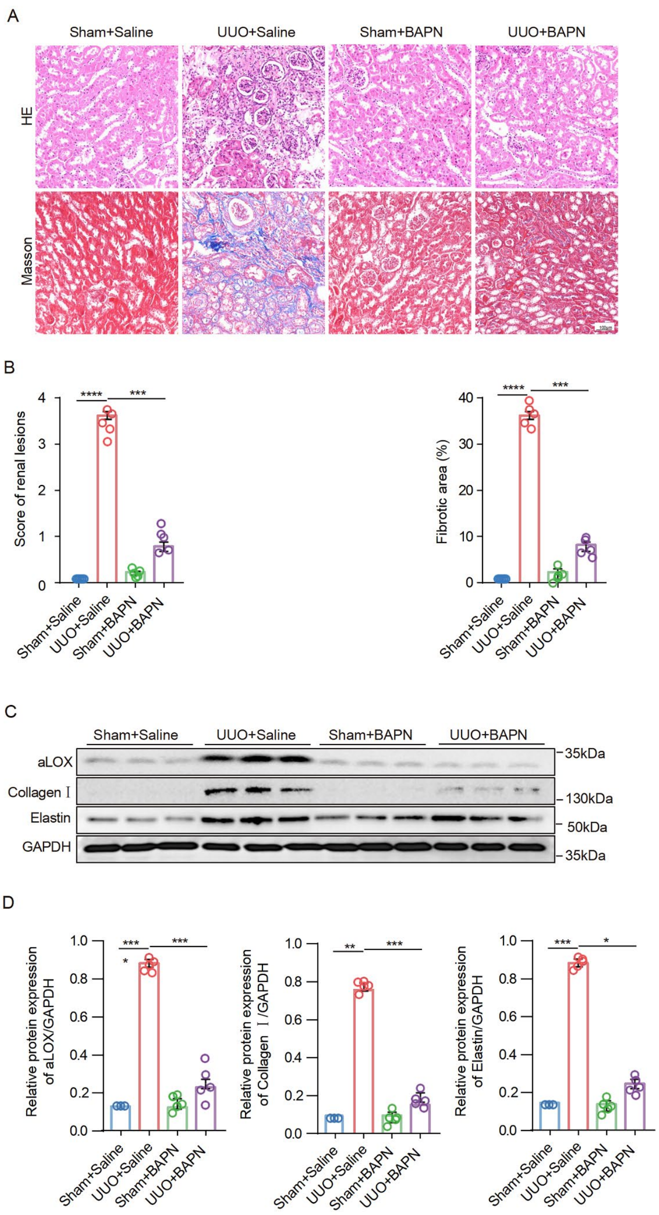

Supplemental Figure 2. Inhibition of LOX activity attenuates renal fibrosis in UUO mice.

(**A-B**) Representative histological images and quantitative analysis of the kidney. H&E staining and Masson's trichrome staining of kidney sections from UUO- mice models (*n* = 5).Original magnification, ×200. Bar = 100 μm.

(**C-D**) Western blot analysis and quantitative analysis of aLOX, Collagen I, and Elastin protein expression in kidney tissues from Sham and UUO mice treated with Saline or BAPN (*n* = 5).

Each bar represents the mean ± SEM, statistics used included 1-way ANOVA. *, $P < 0.05$, **, $P < 0.01$, ***, $P < 0.001$, ****, $P < 0.0001$.

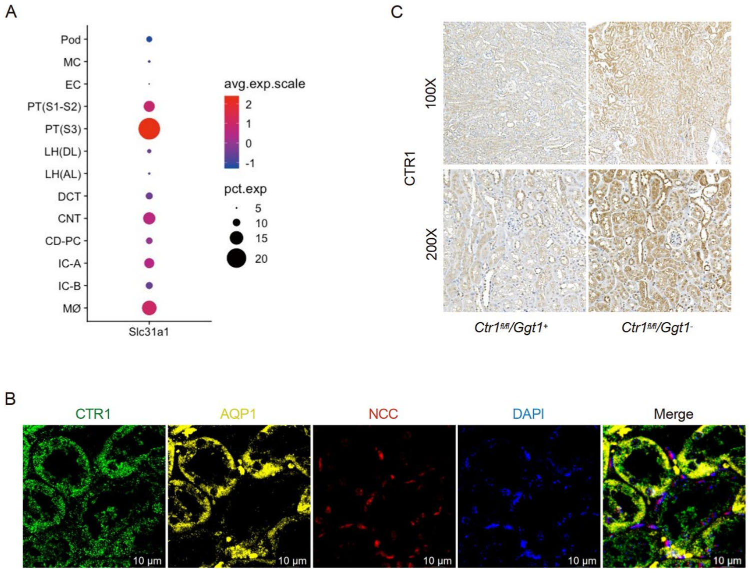

Supplemental Figure 3. The expression of CTR1 in normal mouse kidney and the identification of $Ctr1^{fl/fl}$/ $Cre^{-/-}$ and $Ctr1^{fl/fl}$/$Cre^{+/+}$ mice.

(**A**) The expression profile of CTR1 across various cell types in the single-cell sequencing data of normal mouse kidneys. The data was obtained from the 'Healthy Mouse Dataset: Wu et al, JASN 2019; and RBK RID: 14-4KBC' in https://humphreyslab.com/SingleCell/.

(**B**) The co-localization of CTR1 (green) with AQP1 (yellow) and NCC (red) was analyzed by immunofluorescent co-staining in renal tissues ($n = 3$). AQP1, Aquaporin-1. NCC, Na-Cl cotransporter. Original magnification, ×400. Bar = 10 μm.

(**C**) Representative images of immunohistochemical staining with CTR1 in kidney sections from $Ctr1^{fl/fl}$/ Cre^{-} and $Ctr1^{fl/fl}$/Cre^{+} mice ($n = 5$).

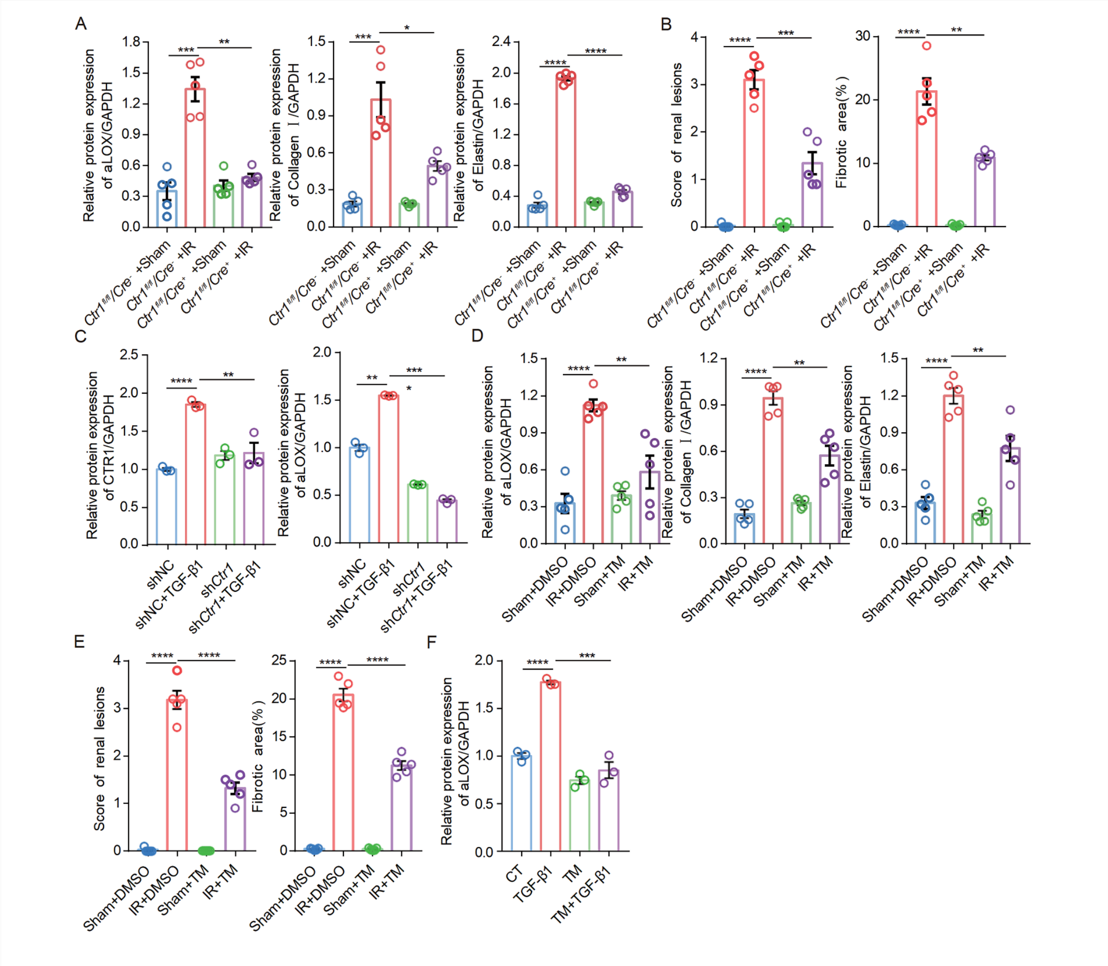

Supplemental Figure 4. The quantitative analysis data of Figure 2 and Figure 3.

For the analyses of *Ctr1* manipulated mice, *Ctr1*$^{fl/fl}$/ *Cre*$^-$ and *Ctr1*$^{fl/fl}$/*Cre*$^+$ mice were randomly divided into the following four groups (n = 5/each group): *Ctr1*$^{fl/fl}$/ *Cre*$^-$ mice + sham; *Ctr1*$^{fl/fl}$/ *Cre*$^-$ mice + IR; *Ctr1*$^{fl/fl}$/*Cre*$^+$ mice + sham; and *Ctr1*$^{fl/fl}$/*Cre*$^+$ mice + IR (**A-B**).

(**A**) Quantitative analysis of the expression of aLOX, Collagen I and Elastin levels detected by Western immunoblots in mouse kidneys.

(**B**) Quantitative analysis of HE and Masson's staining of kidney sections. Original magnification, ×200. Bar = 100 μm.

(**C**) Quantitative analysis of aLOX and CTR1 detected by Western immunoblots in NRK-52E cells treated with TGF-β1 after downregulation of *Ctr1* (n = 3).

For TM treatment, four groups of C57BL/6 mice were prepared (n = 5/each group): Sham + DMSO; Sham + TM (30 mg/kg); IR + DMSO; and IR + TM (**D-E**).

(**D**) Quantitative analysis of aLOX, Collagen I and Elastin levels detected by Western immunoblots in mouse kidneys.

(**E**) Quantitative analysis of HE and Masson's staining of kidney sections. Original magnification, ×200. Bar = 100 μm.

(**F**) Quantitative analysis of aLOX detected by Western immunoblots in NRK-52E cells treated with or without TM after stimulated with TGF-β1 ($n = 3$).

Each bar represents the mean ± SEM, statistics used included 1-way ANOVA. *, $P < 0.05$, **, $P < 0.01$, ***, $P < 0.001$, ****, $P < 0.0001$.

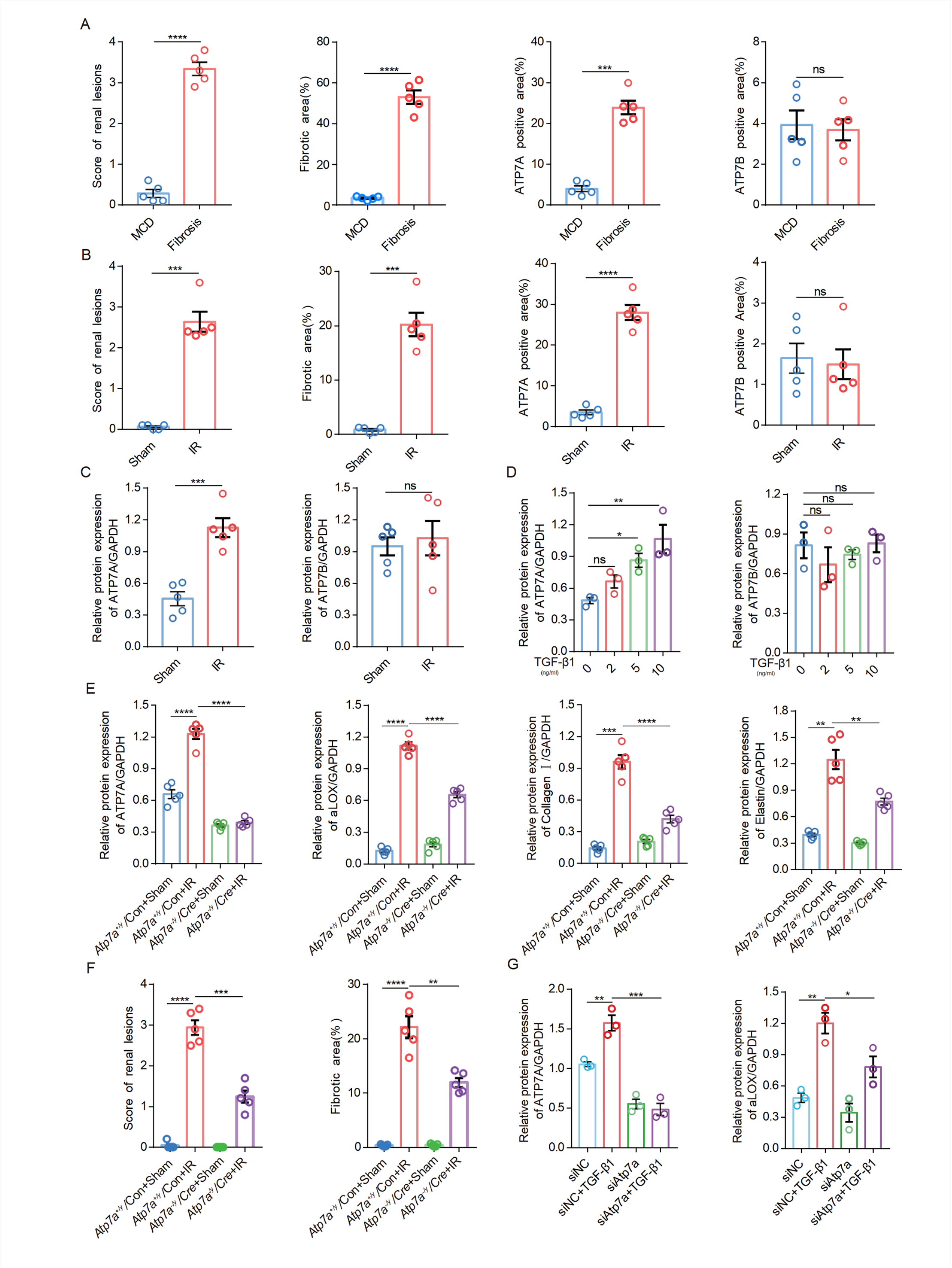

Supplemental Figure 5. The quantitative analysis data of Figure 4 and Figure 5.

(**A**) Quantitative analysis of immunohistochemical staining with ATP7A and ATP7B, HE and Masson's staining in kidney sections from patients with and without renal fibrosis ($n = 5$). Original magnification, ×200. Bar = 100 μm.

(**B**) Quantitative analysis of immunohistochemical staining of ATP7A and ATP7B, HE and Masson's staining in kidney sections from renal fibrosis models induced by IR ($n = 5$). Original magnification,

×200. Bar = 100 μm.

(**C**) Quantitative analysis of ATP7A and ATP7B expression in mouse kidneys detected by Western immunoblots (n = 5).

(**D**) Quantitative analysis of ATP7A and ATP7B expression detected by Western immunoblots in NRK-52E cells treated with TGF-β1 (n = 3).

(**E**) Quantitative analysis ATP7A, aLOX, Collagen I and Elastin expression detected by Western immunoblots in mouse kidneys among different groups (n = 5).

(**F**) Quantitative analysis of HE and Masson's staining of kidney sections (n = 5). Original magnification, ×200. Bar = 100 μm.

(**G**) Quantitative immunoblots of ATP7A and aLOX detected by Western immunoblots in NRK-52E cells among different groups (n = 3).

Each bar represents the mean ± SEM, statistics used included a 2-tailed t test (2 groups, in A-C) or 1-way ANOVA (multiple groups, in D-G). *, $P < 0.05$, **, $P < 0.01$, ***, $P < 0.001$, ****, $P < 0.0001$.

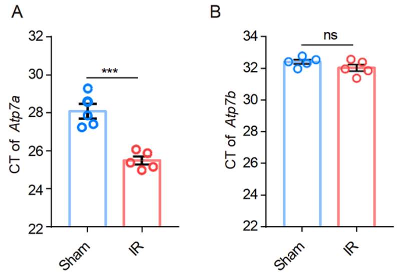

Supplemental Figure 6. The RT-qPCR analysis of *Atp7a* (A)and *Atp7b* (B) mRNA in in kidney subjected by IR injury. CT: cycle threshold. (n = 5), Each bar represents the mean ± SEM, statistics used included a 2-tailed t test. *, $P < 0.05$, **, $P < 0.01$, ***, $P < 0.001$.

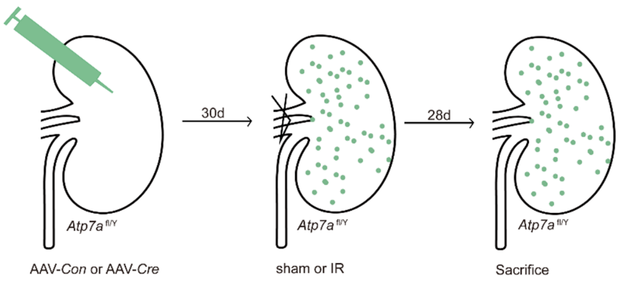

Supplemental Figure 7. The time points for AAV injection and IR or Sham surgery.

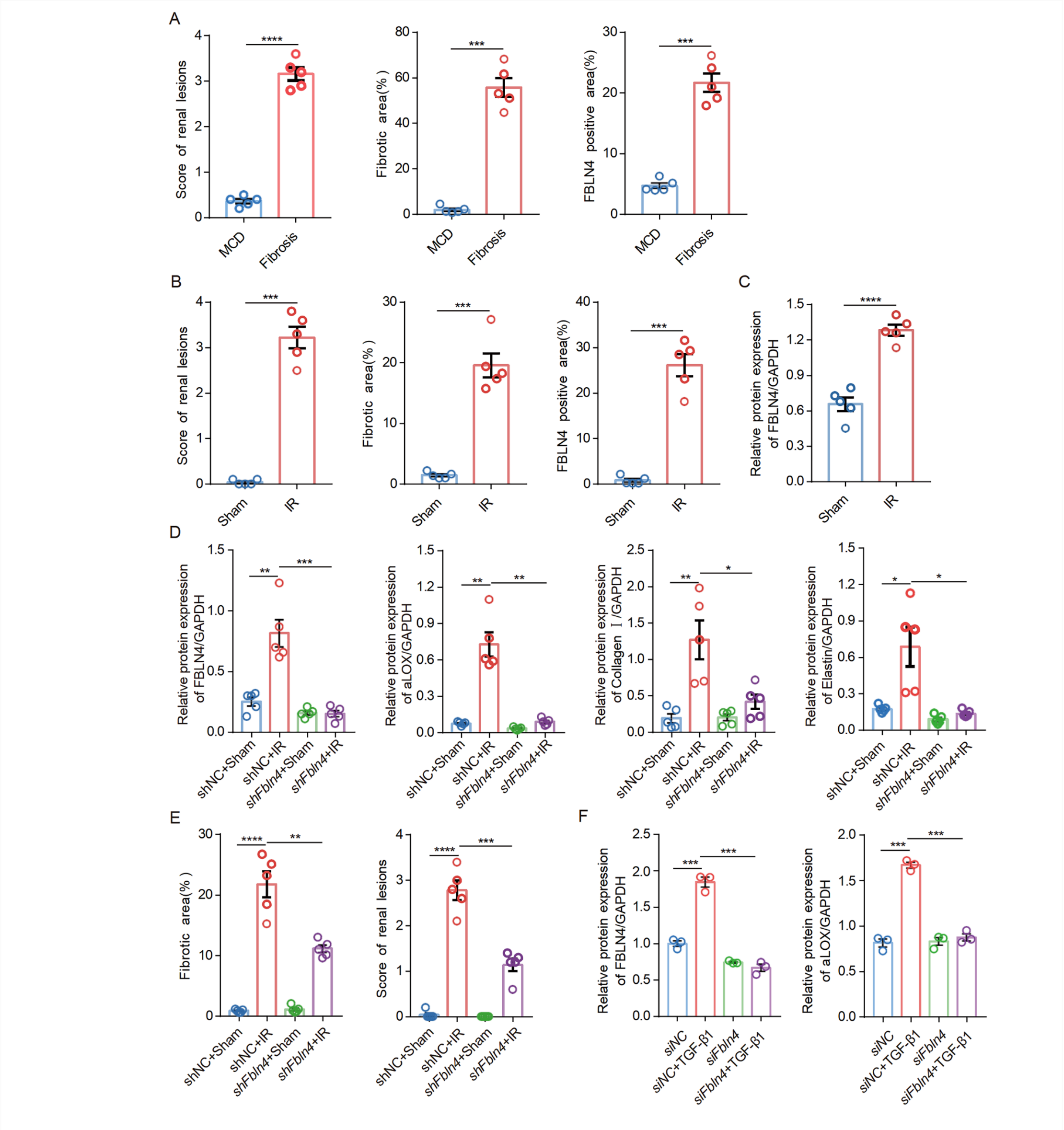

Supplemental Figure 8. The quantitative analysis data of Figure 8.

(**A**) Quantitative analysis of immunohistochemical staining with FBLN4, HE and Masson's staining in kidney sections from patients with and without renal fibrosis (*n* = 5). Original magnification, ×200. Bar = 100 μm.

(**B**) Quantitative analysis of immunohistochemical staining with FBLN4, HE and Masson's staining in kidney subjected by IR injury (*n* = 5). Original magnification, ×200. Bar = 100 μm.

(**C**) Quantitative analysis of FBLN4 detected by Western immunoblots in kidney tissues among different groups (*n* = 5).

All mice were randomly divided into four groups (n = 5 / each group): sham-operated mice treated with AAV9-Con (shNC + Sham), sham-operated mice treated with AAV9-sh*Fbln4* (sh *Fbln4* +

Sham), IR mice treated with AAV9-Con (shNC + IR) and IR mice treated with AAV9- sh *Fbln4* (sh *Fbln4* + IR) (**D-E**).

(**D**) Quantitative analysis of FBLN4, aLOX, Collagen I and Elastin expression detected by Western immunoblots in mouse kidneys among different groups.

(**E**) Quantitative analysis of HE and Masson's staining of kidney sections. Original magnification, ×200. Bar = 100 μm.

(**F**) Quantitative analysis of FBLN4 and aLOX detected by Western immunoblots in NRK-52E cells among different groups ($n = 3$).

Each bar represents the mean ± SEM, statistics used included a 2-tailed t test (2 groups, in A-C) or 1-way ANOVA (multiple groups, in D-F). *, $P < 0.05$, **, $P < 0.01$, ***, $P < 0.001$, ****, $P < 0.0001$.

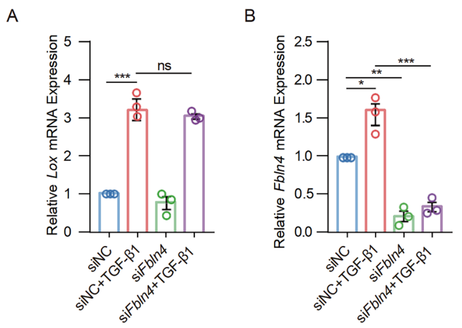

Supplemental Figure 9. The RT-qPCR analysis of *Lox* and *Fbln4* mRNA expression.

(**A-B**) RT-qPCR analysis of *Lox* and *Fbln4* mRNA expression in NRK-52E cells transfected with siNC or si*Fbln4* and treated with or without TGF-β1 ($n = 3$).

Each bar represents the mean ± SEM, statistics used included 1-way ANOVA. *, $P < 0.05$, **, $P < 0.01$, ***, $P < 0.001$, ****, $P < 0.0001$.

Name	Forward Primer	Reverse Primer
Gapdh-mouse	5' AAGGTCGGTGTGAACGGATT 3'	5'CGCTCCTGGAAGATGGTGAT 3'
Atp7a -mouse	5' TGGGAAAGTGAATGGTGTCCA 3'	5'ACGGTATTGGTTAAGACAGGGA3'
Atp7b-mouse	5' GGGGACGATGCCTGAACAG 3'	5' GCCGGGCAAAGCAAGTTTAG 3'
Gapdh-rat	5' GGCAAGTTCAACGGCACA 3'	5' CCATTTGATGTTAGCGGGAT 3'
Fbln4-rat	5' AGACATTGACGAGTGCGAAACAG 3'	5' AGGACAGAGGCAGCGGTTG 3'
Lox-rat	5' GTTCAGCATATAGGGCGGATGTCAG 3'	5' GGCGGCTTGGTAAGAAGTCAG AC 3'

Table 1 Sequences of the primer pairs used for real-time PCR.
