# Supplementary material for: ATP7A-fibulin-4 complex delivers copper in the Golgi to activate LOX in renal fibrosis
Source: JCI Insight. 2026 May 8;11(9):e199028. doi: 10.1172/jci.insight.199028 (PMC13232011; doi:10.1172/jci.insight.199028)

Original data
-Western Blot

Figure 1E

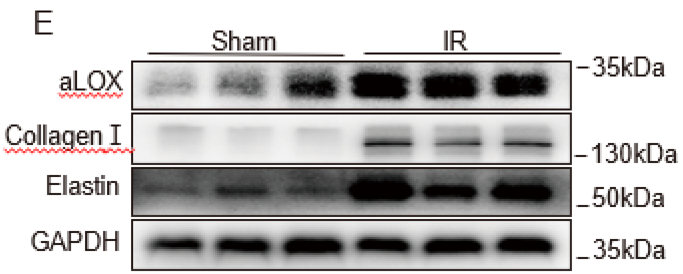
E
Sham
IR
aLOX
Collagen I
Elastin
GAPDH
35kDa
130kDa
50kDa
35kDa

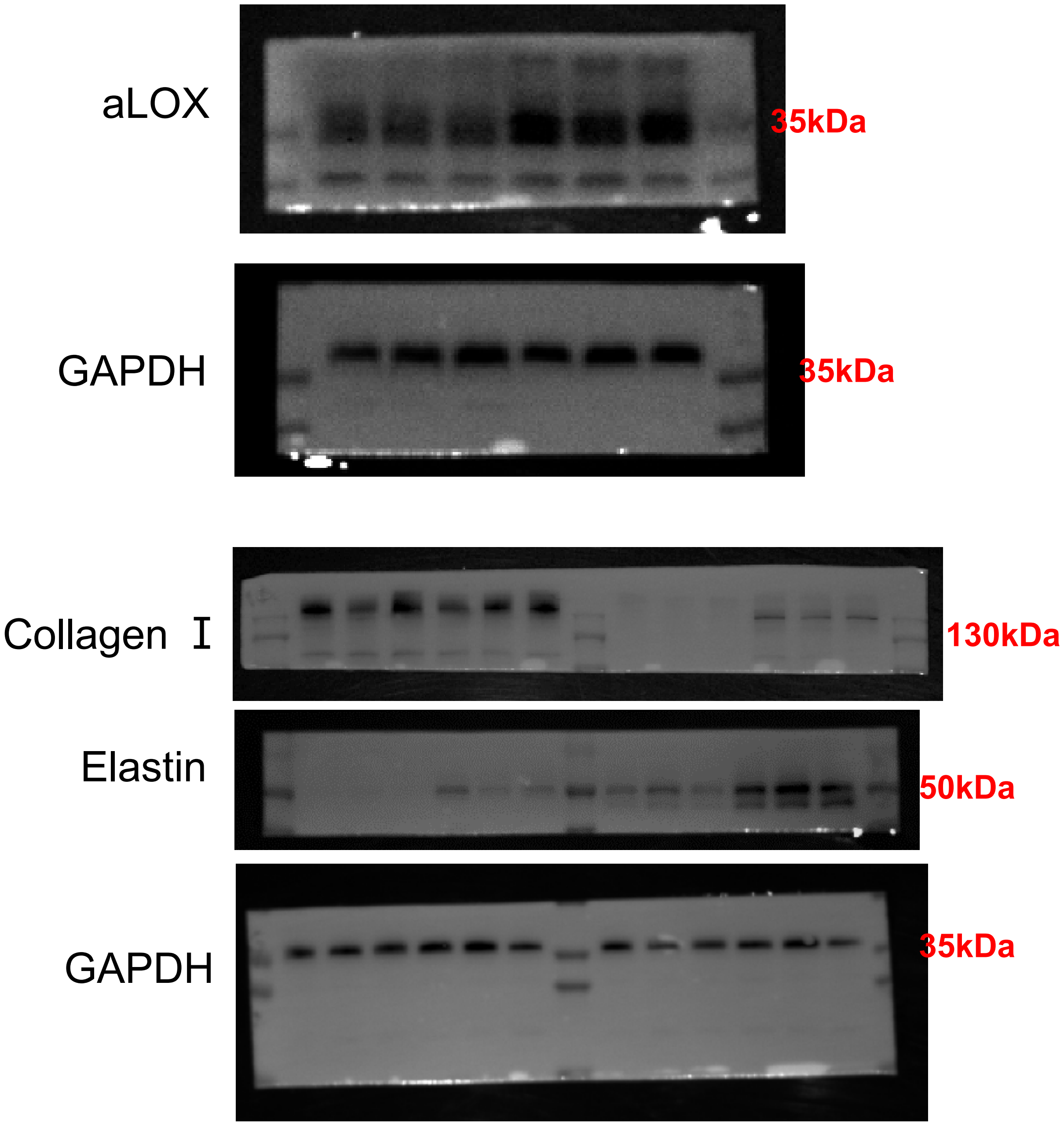
aLOX
35kDa
GAPDH
35kDa
Collagen I
130kDa
Elastin
50kDa
GAPDH
35kDa

Figure 1F

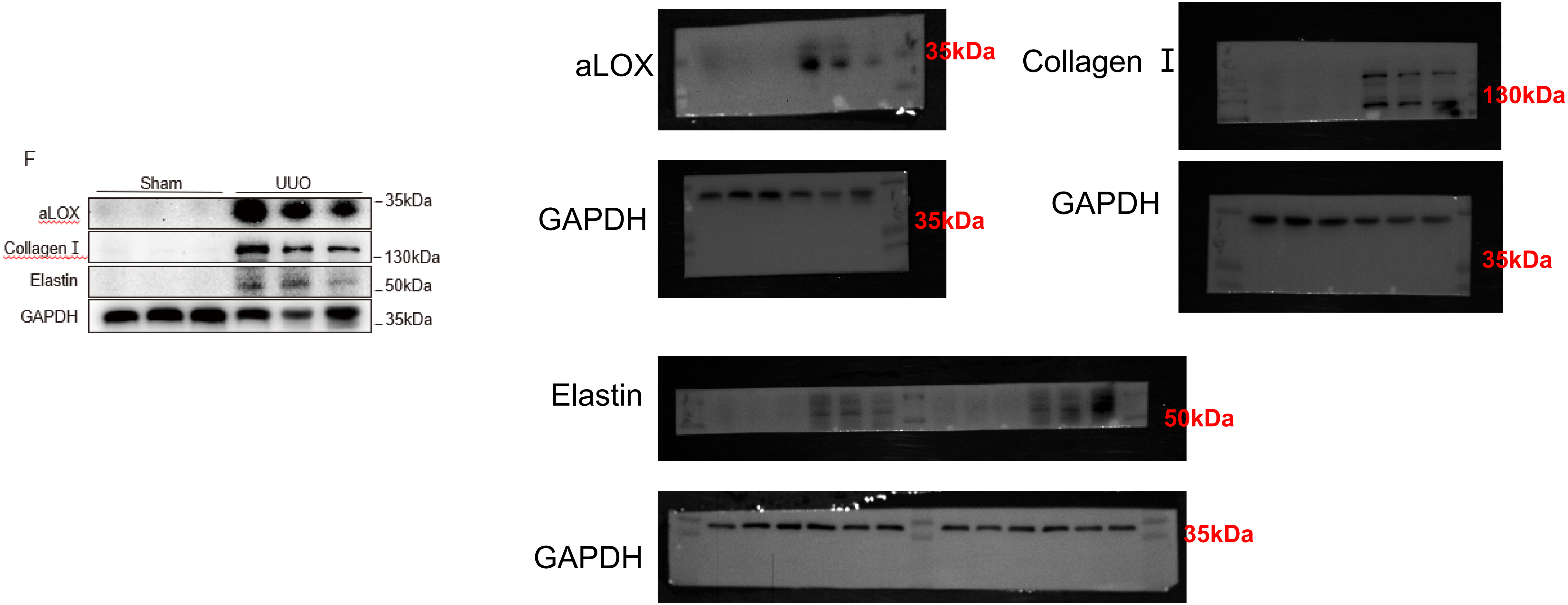
F
Sham
UUO
aLOX
Collagen I
Elastin
GAPDH
35kDa
130kDa
50kDa
35kDa
aLOX
35kDa
Collagen I
130kDa
GAPDH
35kDa
GAPDH
35kDa
Elastin
50kDa
GAPDH
35kDa

Figure 1G

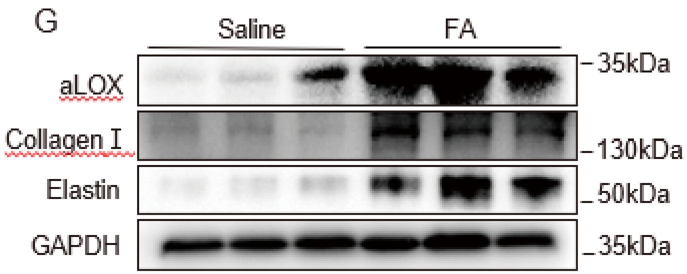
G
Saline
FA
aLOX
Collagen I
Elastin
GAPDH
35kDa
130kDa
50kDa
35kDa

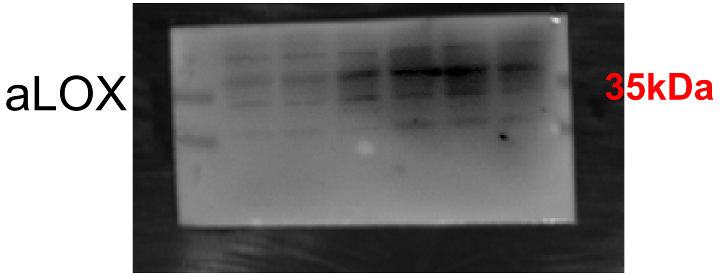
aLOX
35kDa

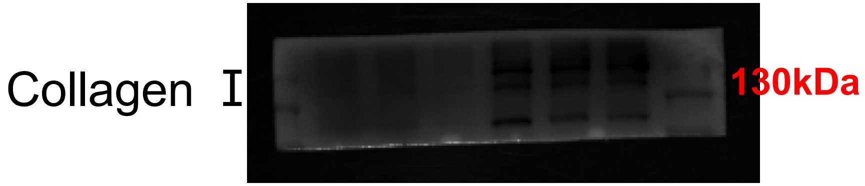
Collagen I
130kDa

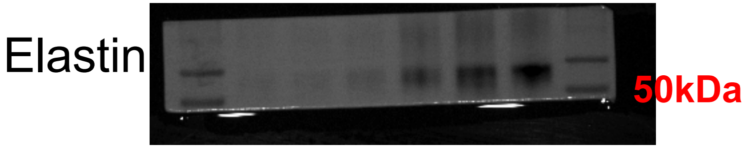
Elastin
50kDa

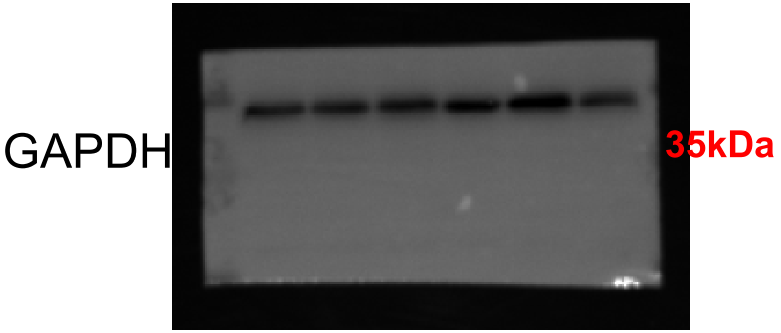
GAPDH
35kDa

Figure 1K

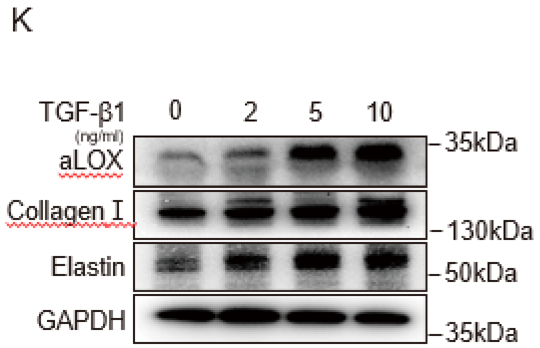
K
TGF-β1
(ng/ml)
0
2
5
10
aLOX
Collagen I
Elastin
GAPDH
35kDa
130kDa
50kDa
35kDa

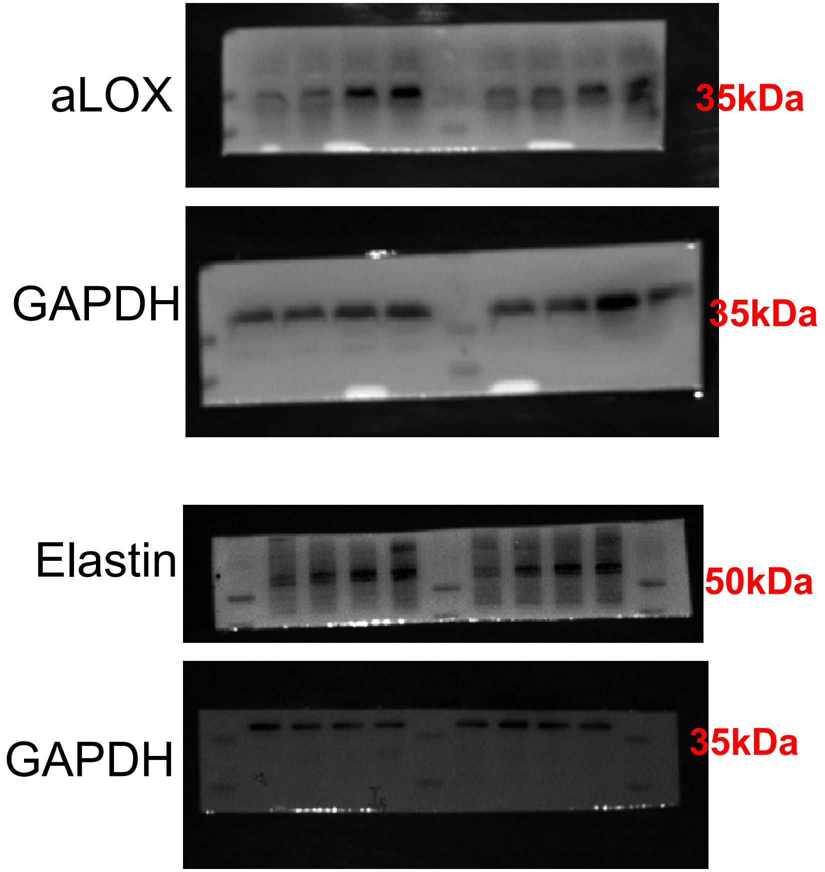
aLOX
35kDa
GAPDH
35kDa
Elastin
50kDa
GAPDH
35kDa

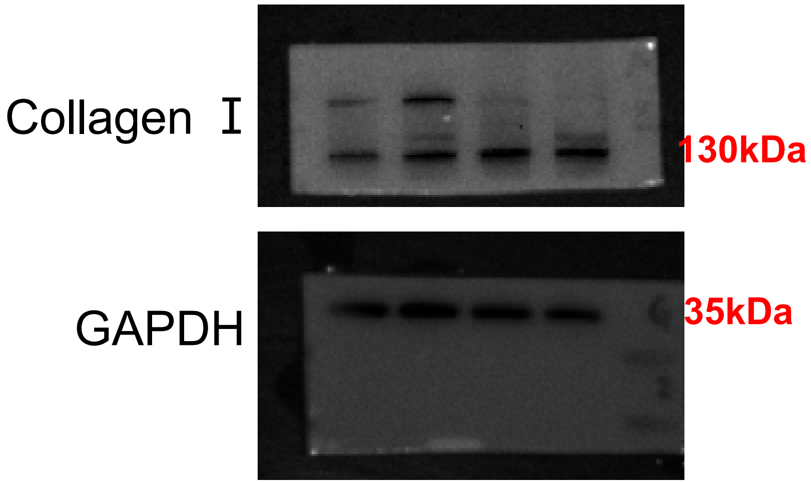
Collagen Ⅰ
130kDa
GAPDH
35kDa

Figure 1M

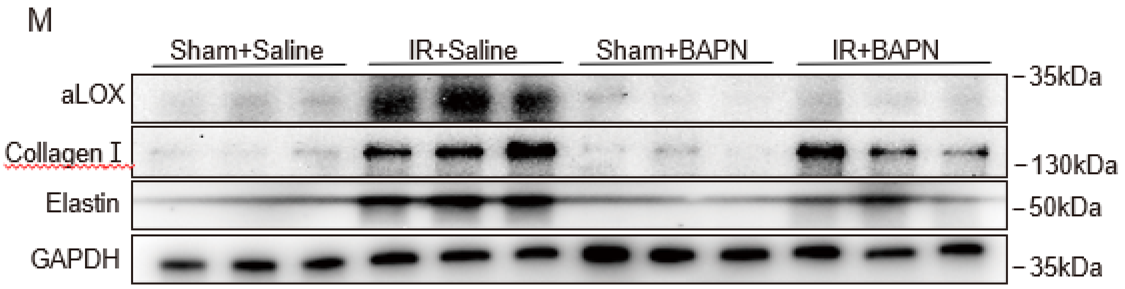

M
Sham+Saline
IR+Saline
Sham+BAPN
IR+BAPN
aLOX
Collagen I
Elastin
GAPDH
35kDa
130kDa
50kDa
35kDa

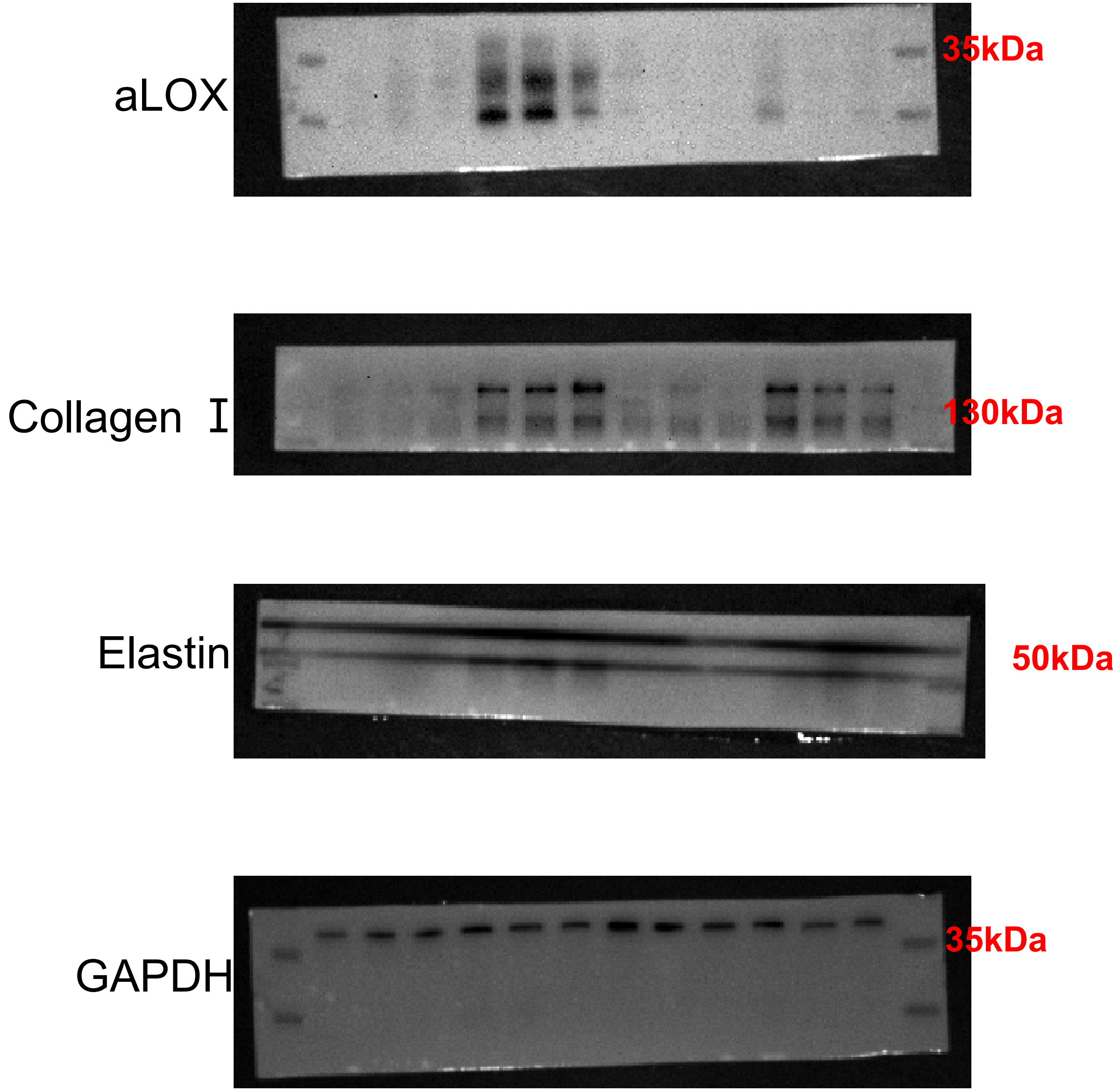

aLOX
35kDa
Collagen I
130kDa
Elastin
50kDa
GAPDH
35kDa

Figure 2D

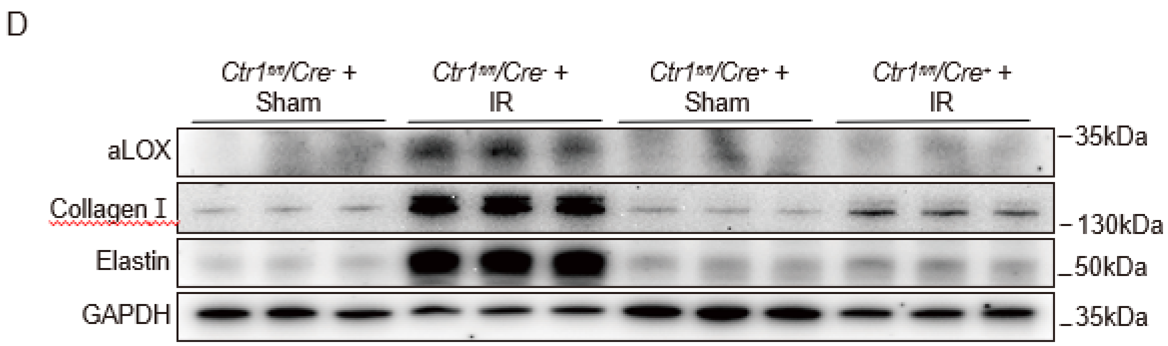
D
Sham
IR
Sham
IR
aLOX
Collagen I
Elastin
GAPDH
35kDa
130kDa
50kDa
35kDa

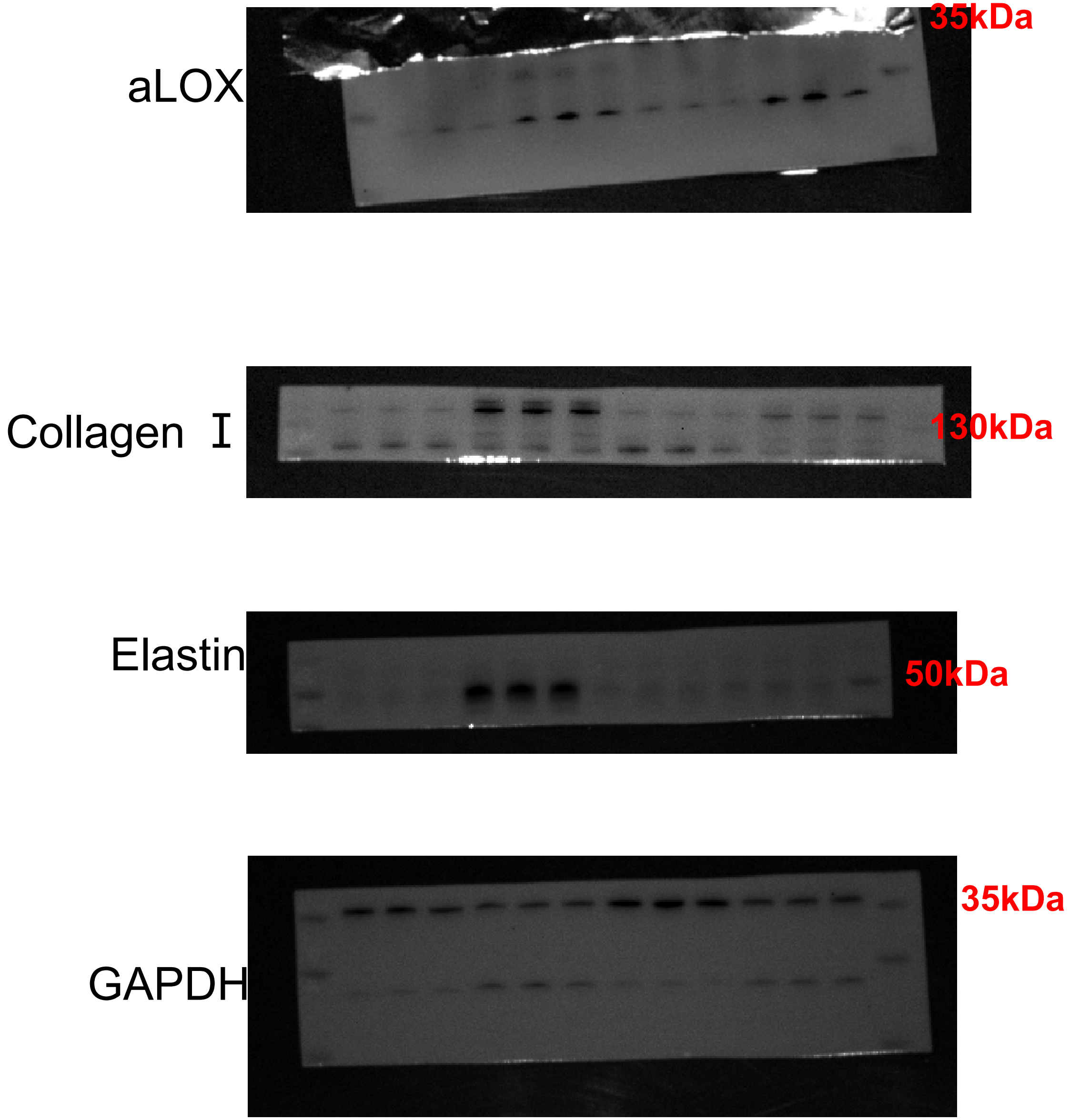
aLOX
35kDa
Collagen I
130kDa
Elastin
50kDa
GAPDH
35kDa

Figure 2J

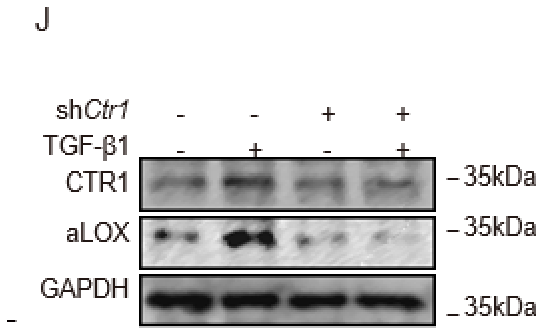
J
shCtr1 - - + +
TGF-β1 - + - +
CTR1
aLOX
GAPDH
35kDa
35kDa
35kDa

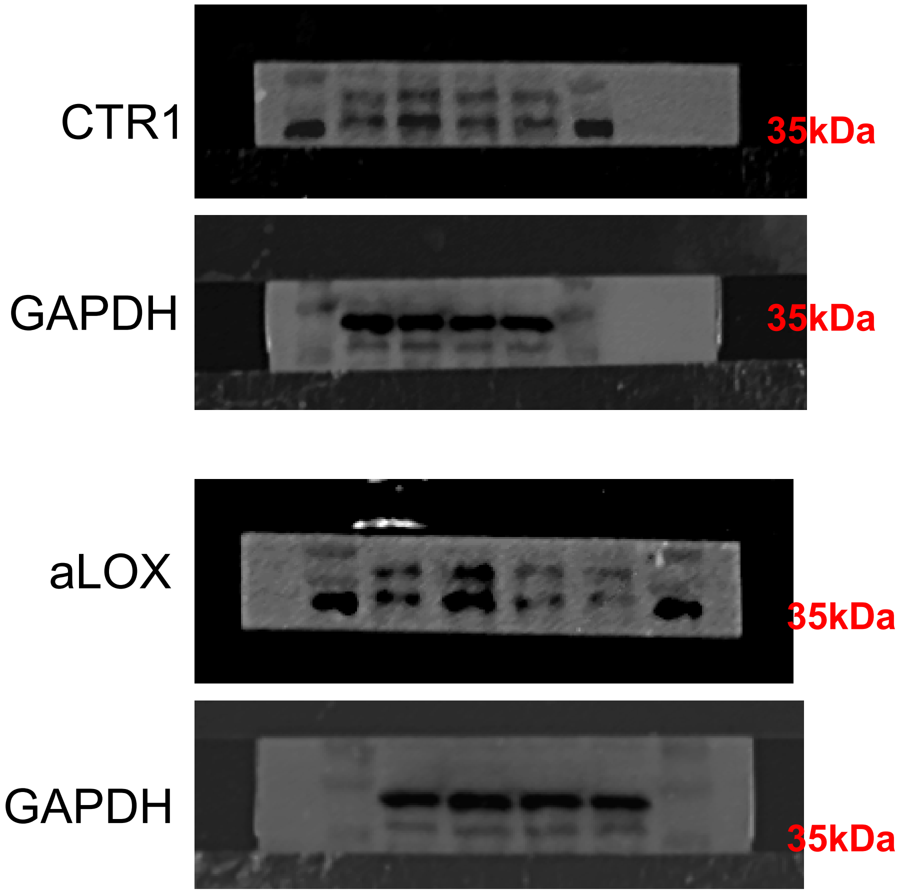
CTR1
35kDa
GAPDH
35kDa
aLOX
35kDa
GAPDH
35kDa

Figure 3C

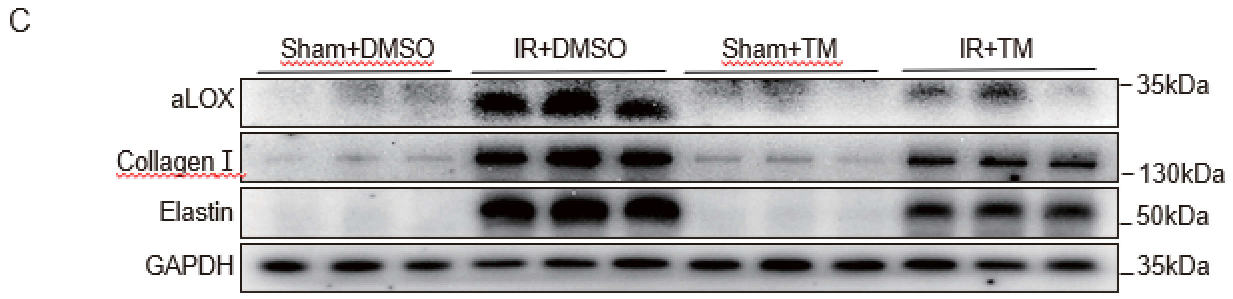
C
Sham+DMSO
IR+DMSO
Sham+TM
IR+TM
aLOX
Collagen I
Elastin
GAPDH
35kDa
130kDa
50kDa
35kDa

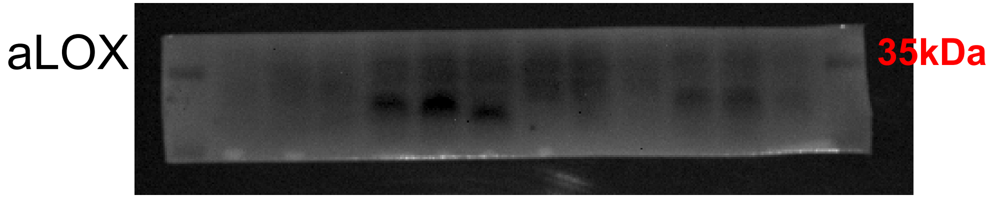
aLOX
35kDa

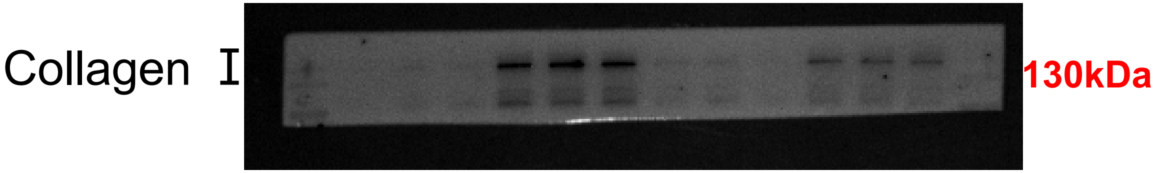
Collagen Ⅰ
130kDa

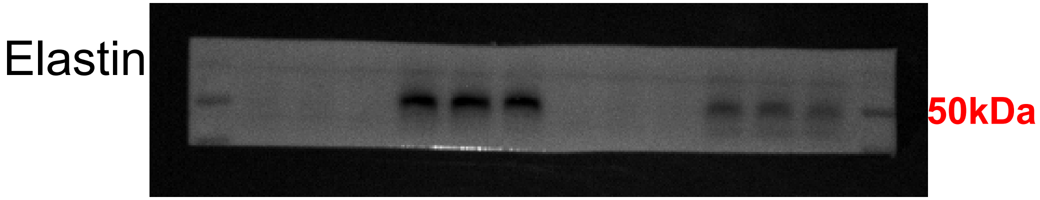
Elastin
50kDa

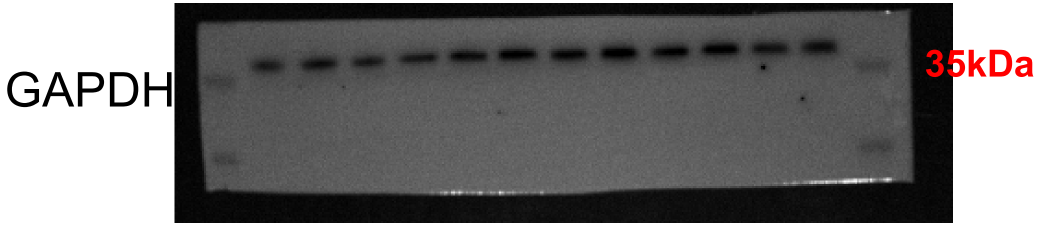
GAPDH
35kDa

Figure 3H

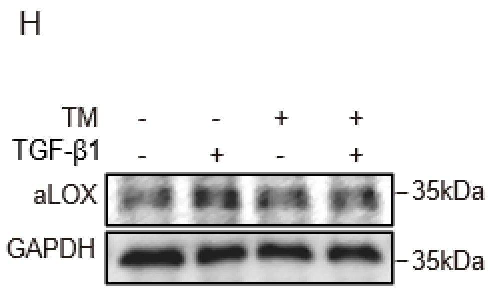

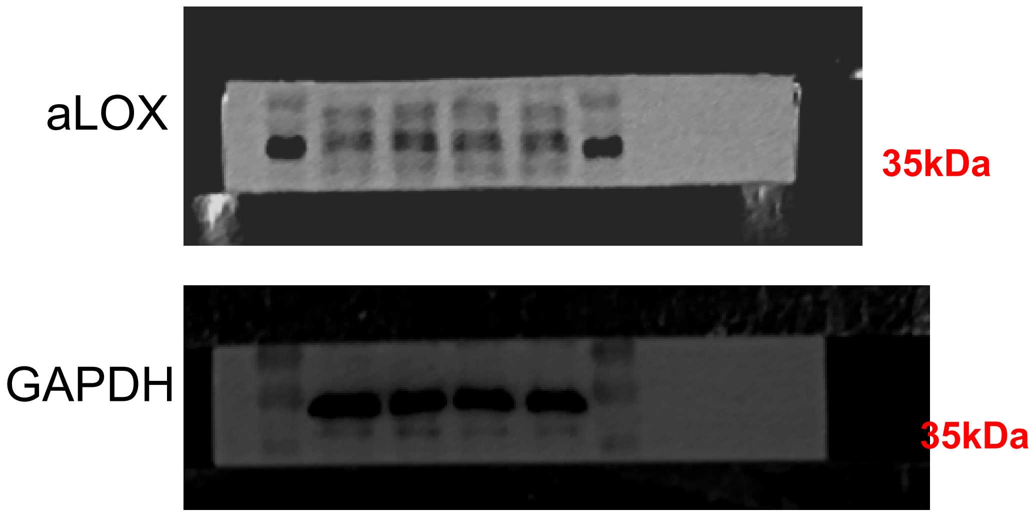

Figure 4C

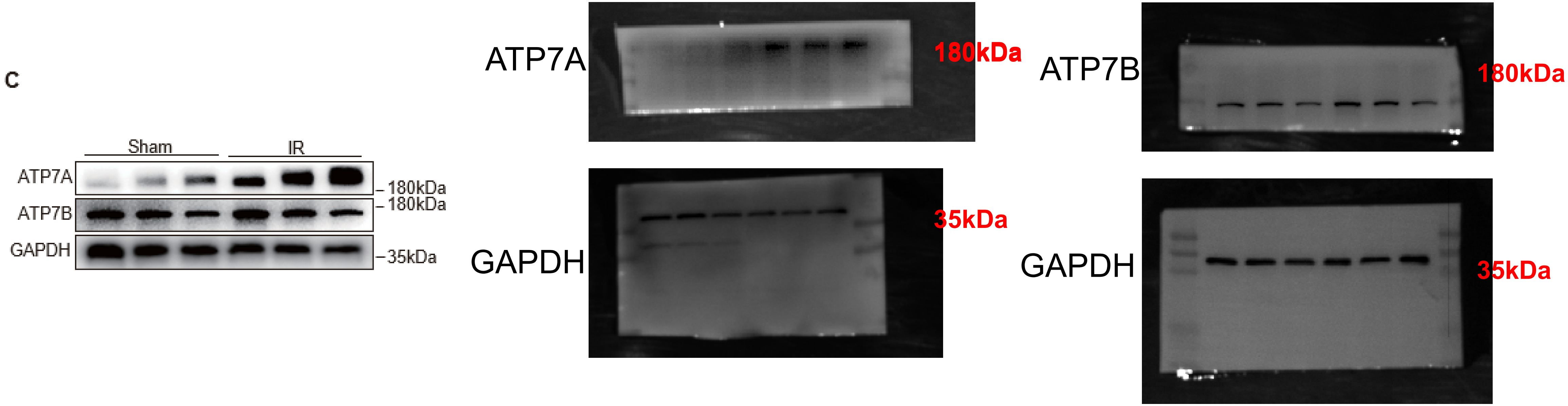
C
Sham
IR
ATP7A
ATP7B
GAPDH
180kDa
180kDa
35kDa
ATP7A
180kDa
GAPDH
35kDa
ATP7B
180kDa
GAPDH
35kDa

Figure 4E

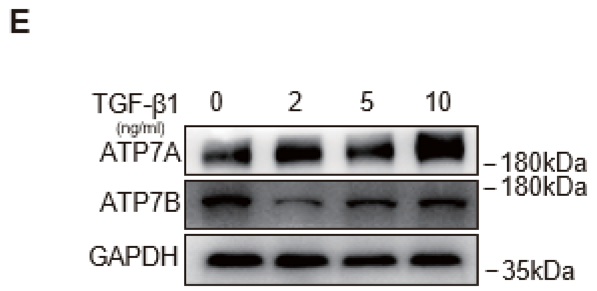
E
TGF-β1
(ng/ml)
0
2
5
10
ATP7A
ATP7B
GAPDH
180kDa
180kDa
35kDa

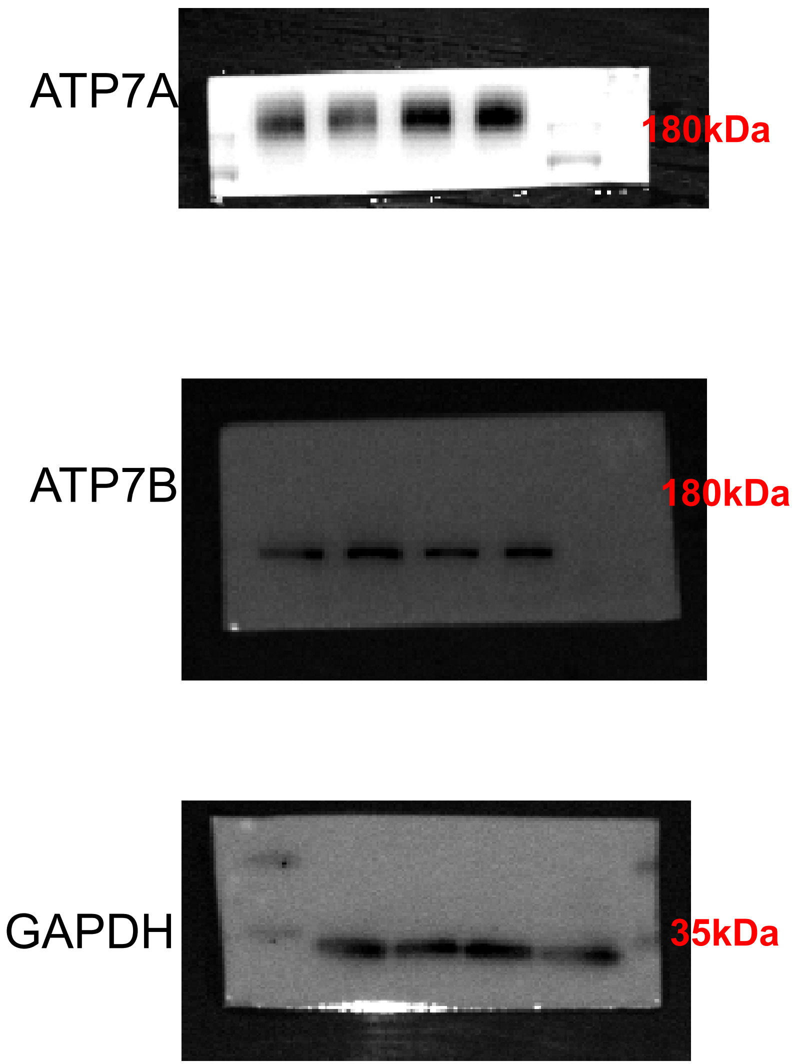
ATP7A
180kDa
ATP7B
180kDa
GAPDH
35kDa

Figure 5C

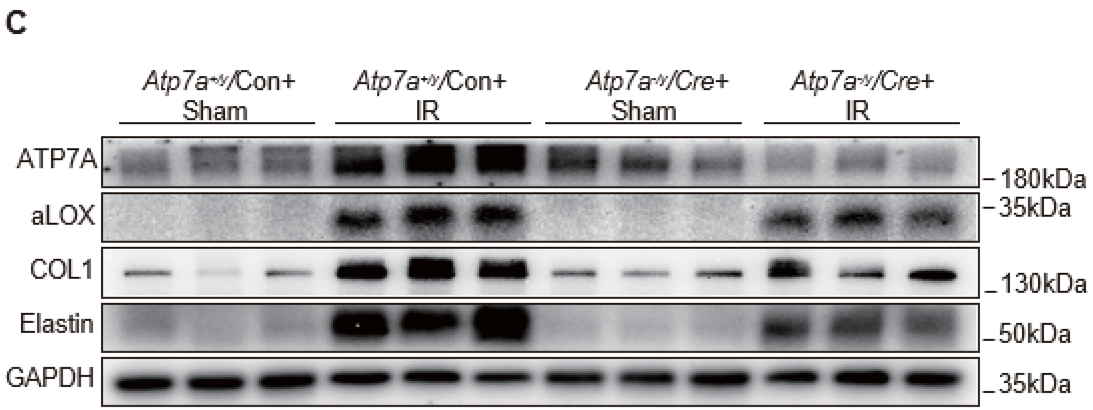
C
Atp7a+/y/Con+
Sham
Atp7a+/y/Con+
IR
Atp7a-/y/Cre+
Sham
Atp7a-/y/Cre+
IR
ATP7A
aLOX
COL1
Elastin
GAPDH
180kDa
35kDa
130kDa
50kDa
35kDa

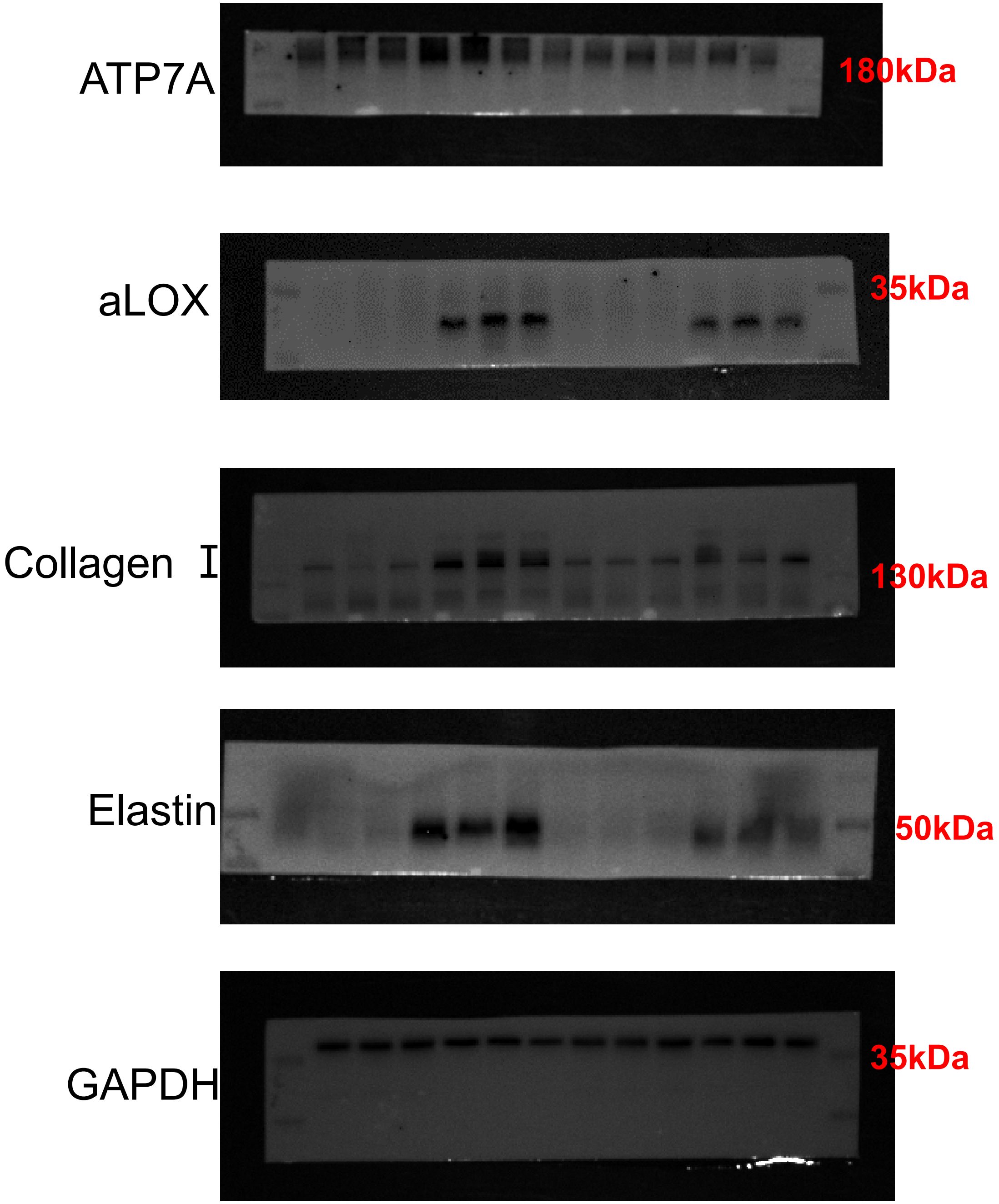
ATP7A
180kDa
aLOX
35kDa
Collagen Ⅰ
130kDa
Elastin
50kDa
GAPDH
35kDa

Figure 5F

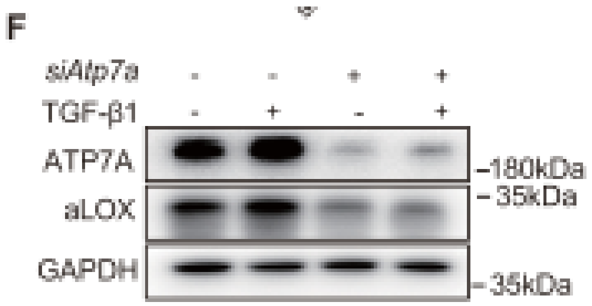
F
siAtp7a - - + +
TGF-β1 - + - +
ATP7A
aLOX
GAPDH
180kDa
35kDa
35kDa

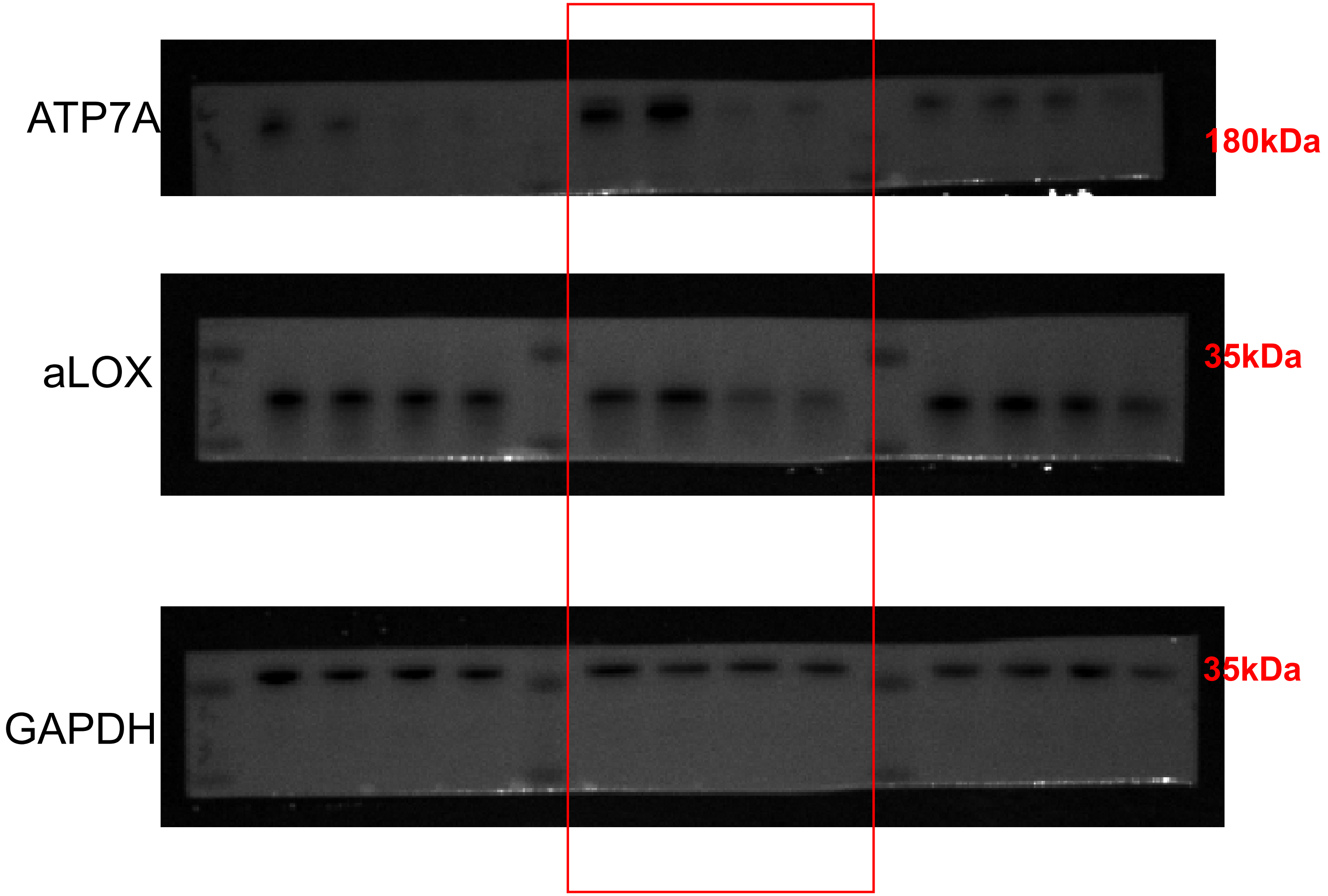
ATP7A
aLOX
GAPDH
180kDa
35kDa
35kDa

Figure 7C

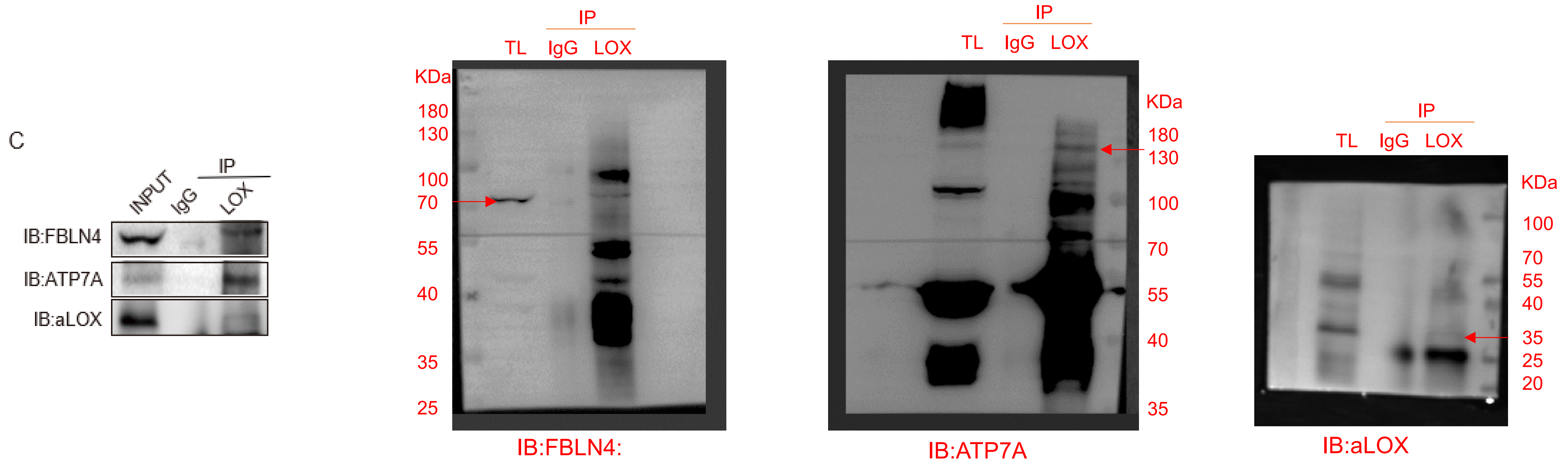

C
INPUT
IgG
LOX
IP
IB:FBLN4
IB:ATP7A
IB:aLOX
TL
IgG
LOX
IP
KDa
180
130
100
70
55
40
35
25
IB:FBLN4:
TL
IgG
LOX
IP
KDa
180
130
100
70
55
40
35
IB:ATP7A
TL
IgG
LOX
IP
KDa
100
70
55
40
35
25
20
IB:aLOX

Figure 7D

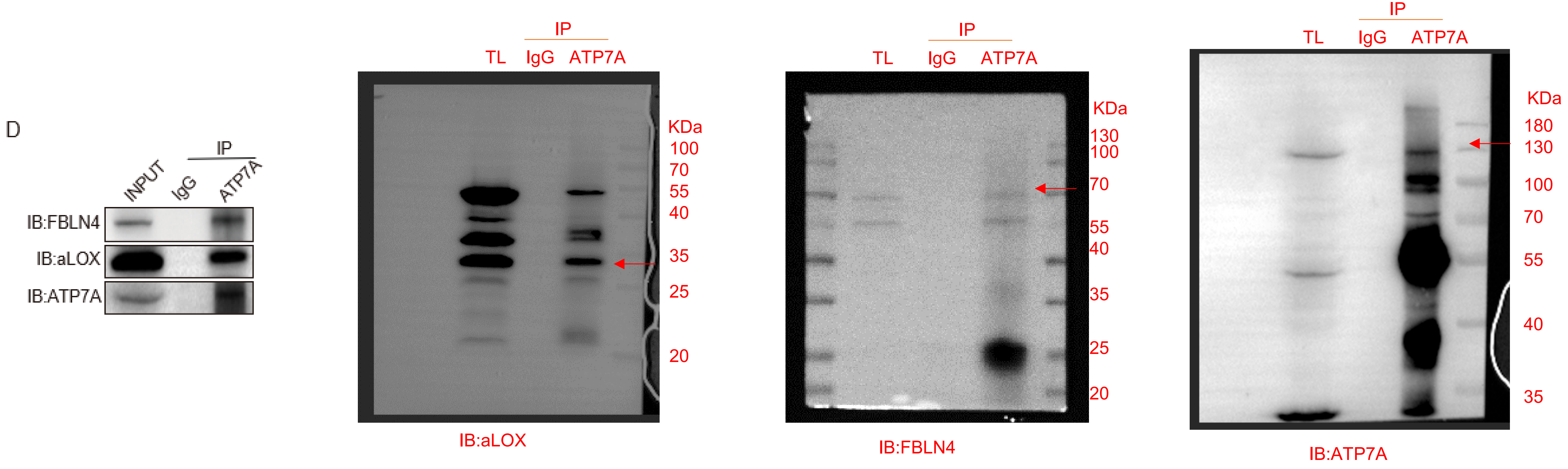
D
IP
INPUT
IgG
ATP7A
IB:FBLN4
IB:aLOX
IB:ATP7A
IP
TL
IgG
ATP7A
KDa
100
70
55
40
35
25
20
IB:aLOX
IP
TL
IgG
ATP7A
KDa
130
100
70
55
40
35
25
20
IB:FBLN4
IP
TL
IgG
ATP7A
KDa
180
130
100
70
55
40
35
IB:ATP7A

Figure 7E

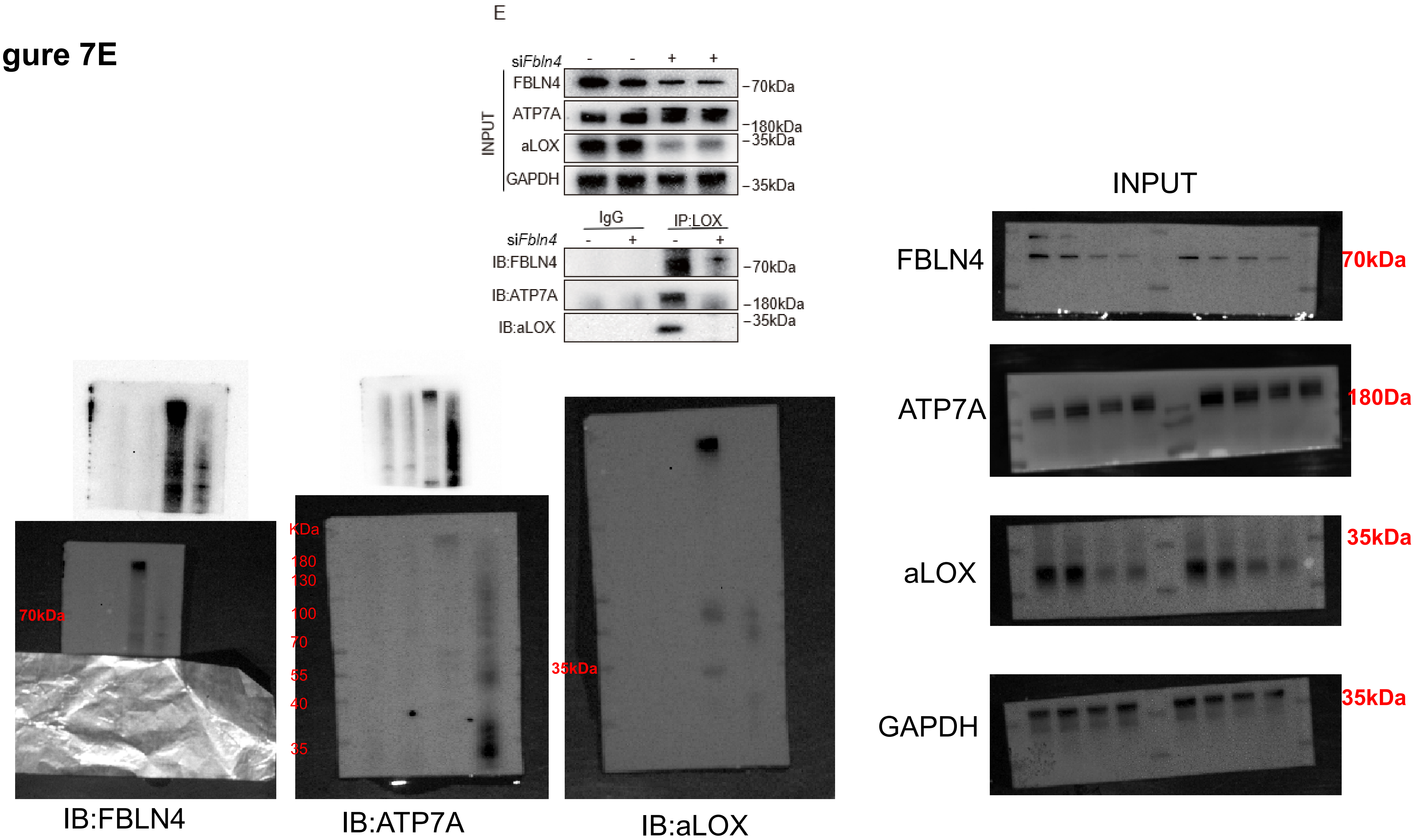
E
siFbln4 - - + +
INPUT
FBLN4
ATP7A
aLOX
GAPDH
70kDa
180kDa
35kDa
35kDa
IgG
IP:LOX
siFbln4 - + - +
IB:FBLN4
IB:ATP7A
IB:aLOX
70kDa
180kDa
35kDa
70kDa
KDa
180
130
100
70
55
40
35
35kDa
IB:FBLN4
IB:ATP7A
IB:aLOX
INPUT
FBLN4
ATP7A
aLOX
GAPDH
70kDa
180Da
35kDa
35kDa

Figure 7F

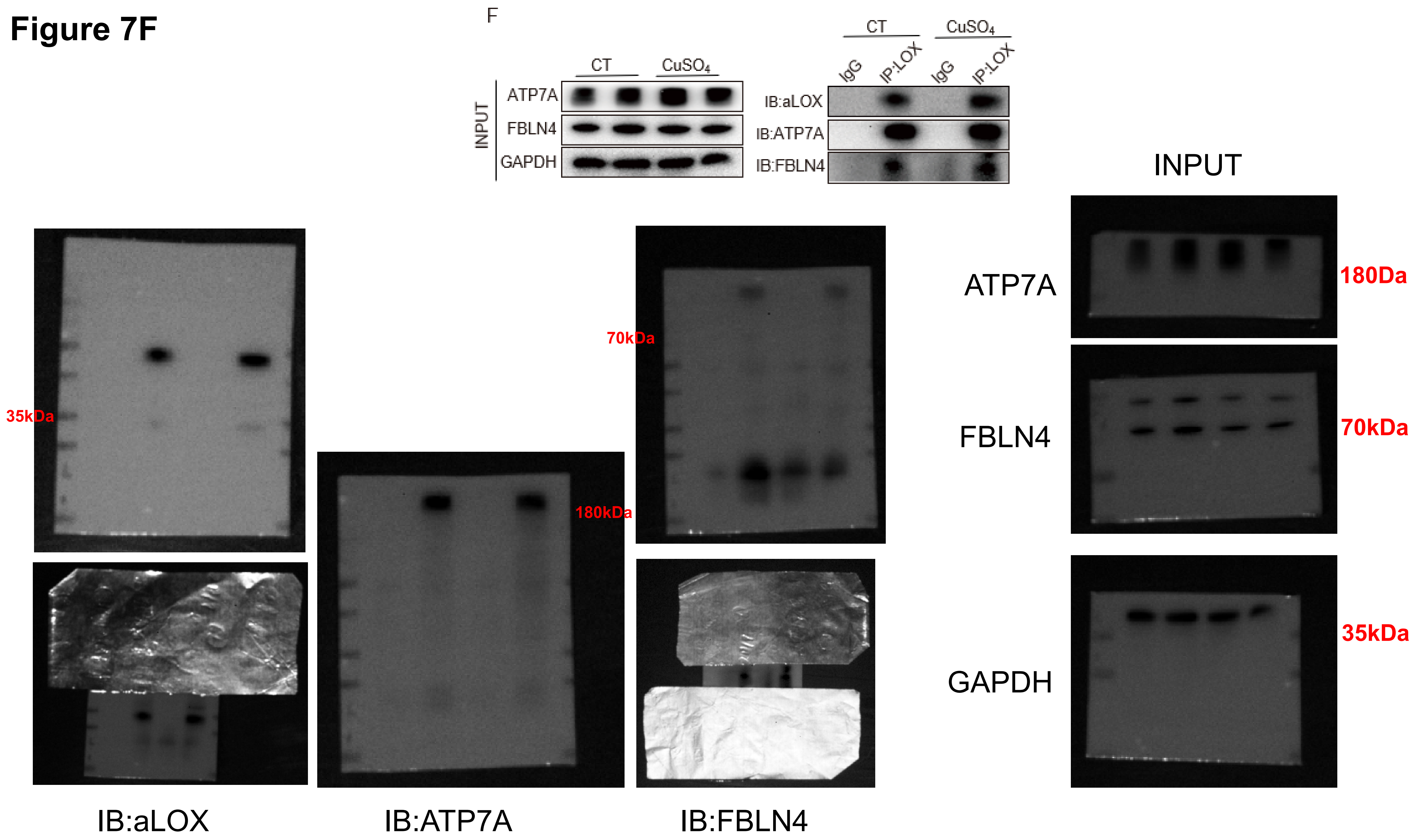

F
INPUT
CT
CuSO4
ATP7A
FBLN4
GAPDH
IgG
IP:LOX
IB:aLOX
IB:ATP7A
IB:FBLN4
35kDa
180kDa
70kDa
INPUT
ATP7A
180Da
FBLN4
70kDa
GAPDH
35kDa
IB:aLOX
IB:ATP7A
IB:FBLN4

Figure 7G

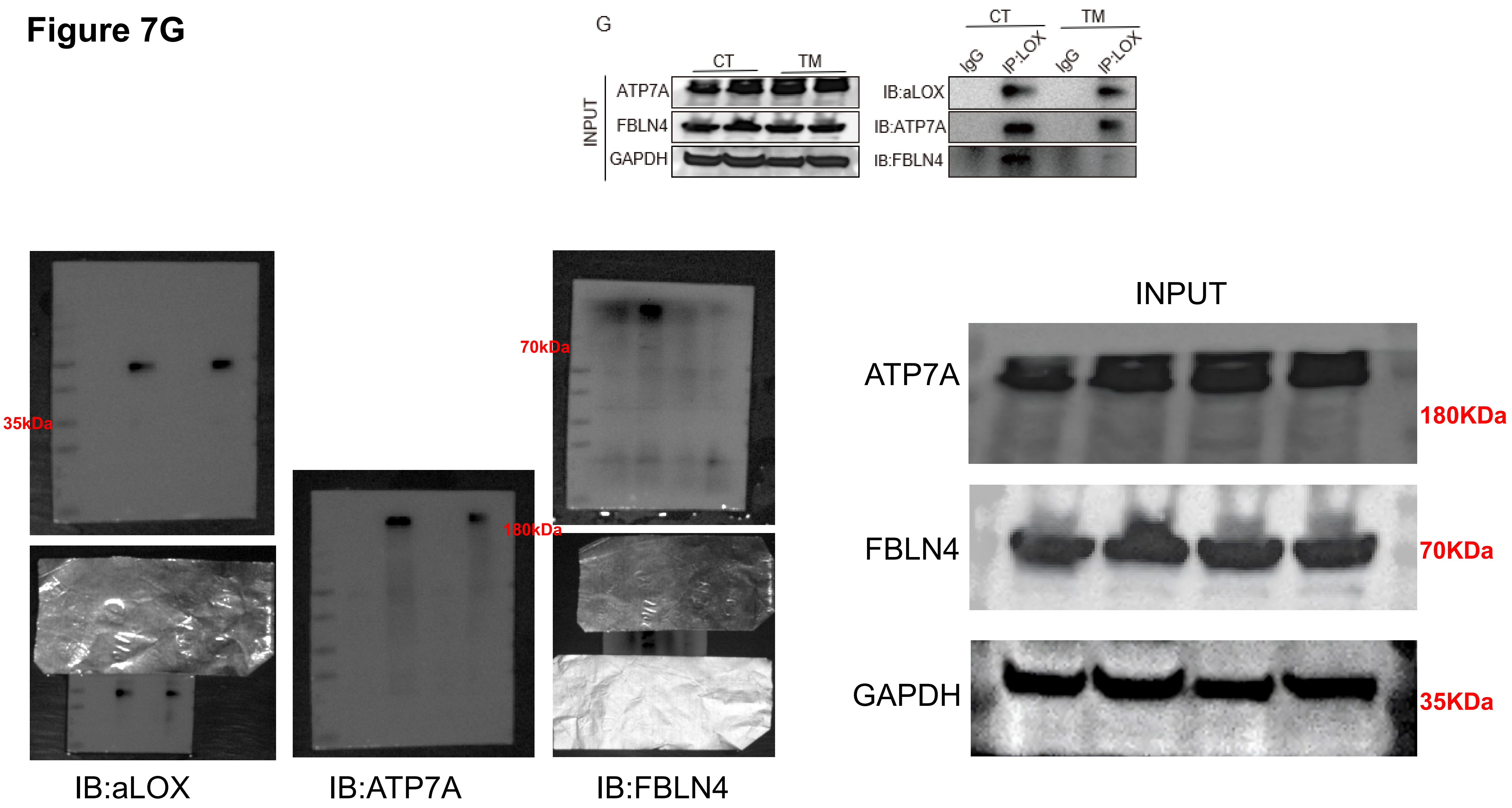
G
CT
TM
INPUT
ATP7A
FBLN4
GAPDH
IgG
IP:LOX
IB:aLOX
IB:ATP7A
IB:FBLN4
35kDa
70kDa
180kDa
INPUT
180KDa
70KDa
35KDa

Figure 8C

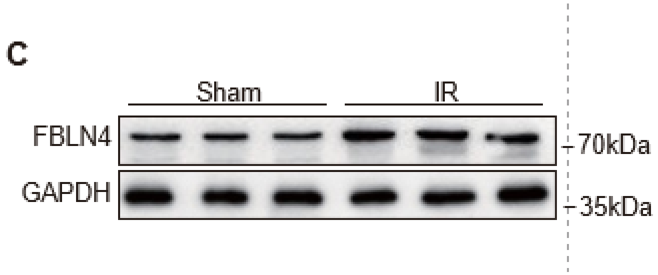

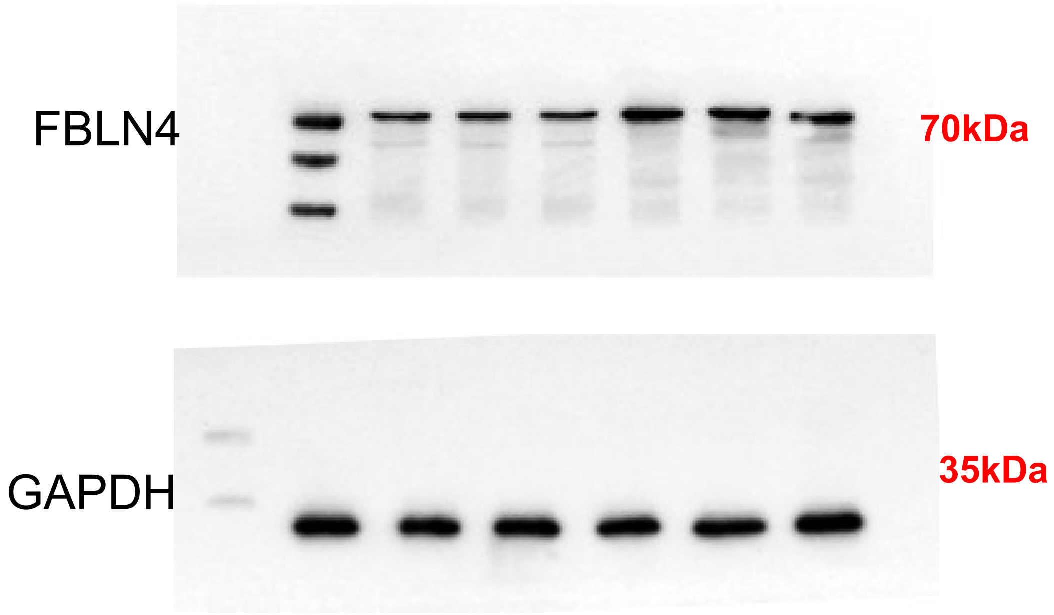

Figure 8E

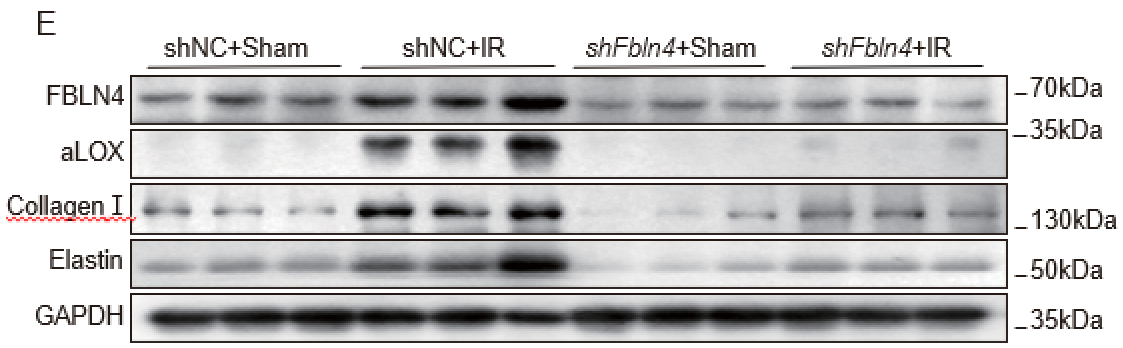

E
shNC+Sham
shNC+IR
shFbln4+Sham
shFbln4+IR
FBLN4
aLOX
Collagen I
Elastin
GAPDH
70kDa
35kDa
130kDa
50kDa
35kDa

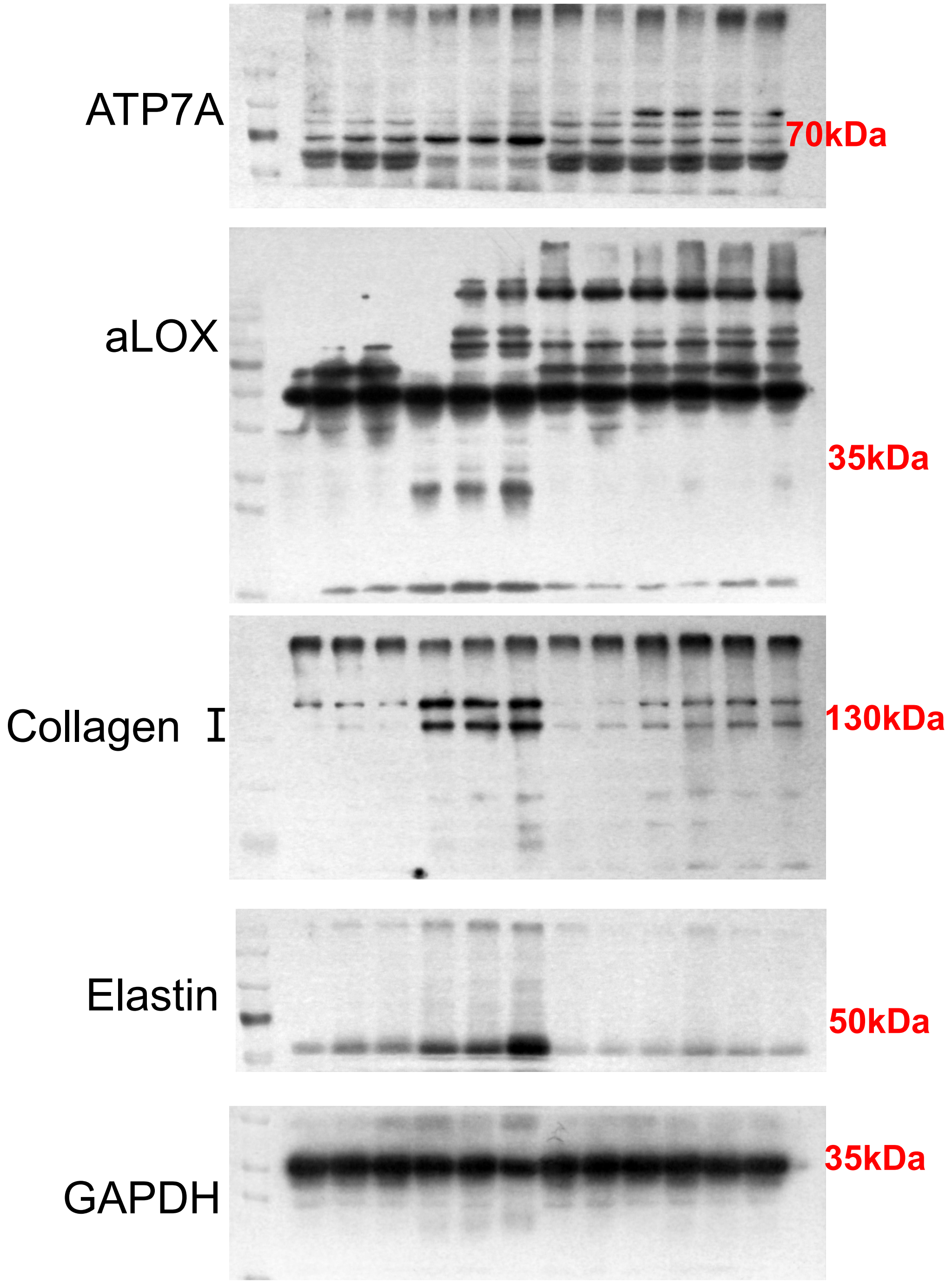

ATP7A
70kDa
aLOX
35kDa
Collagen I
130kDa
Elastin
50kDa
GAPDH
35kDa

Figure 8H

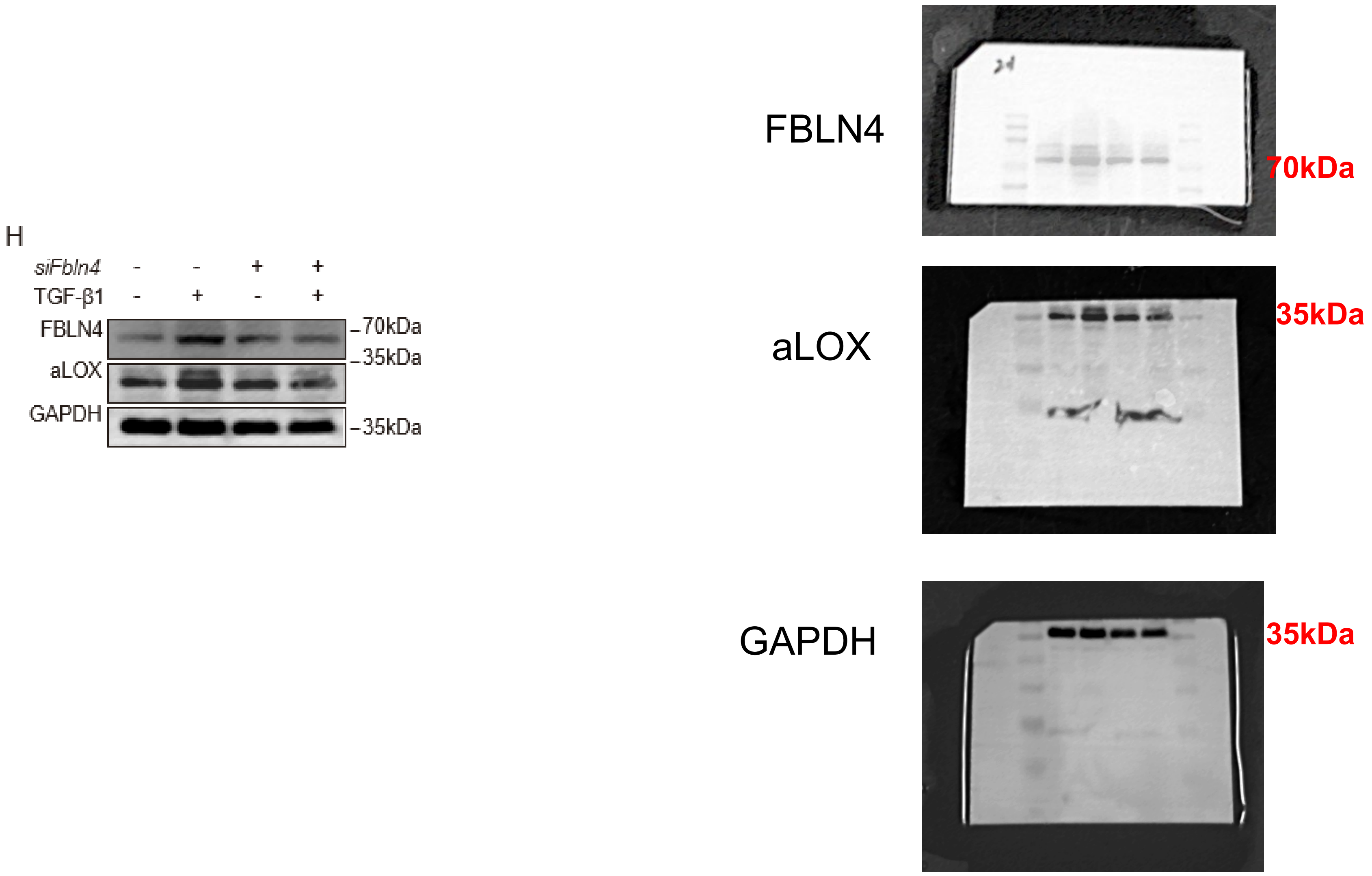
H
siFbln4 - - + +
TGF-β1 - + - +
FBLN4
aLOX
GAPDH
70kDa
35kDa
35kDa
FBLN4
70kDa
aLOX
35kDa
GAPDH
35kDa

Supplement Figure 2C

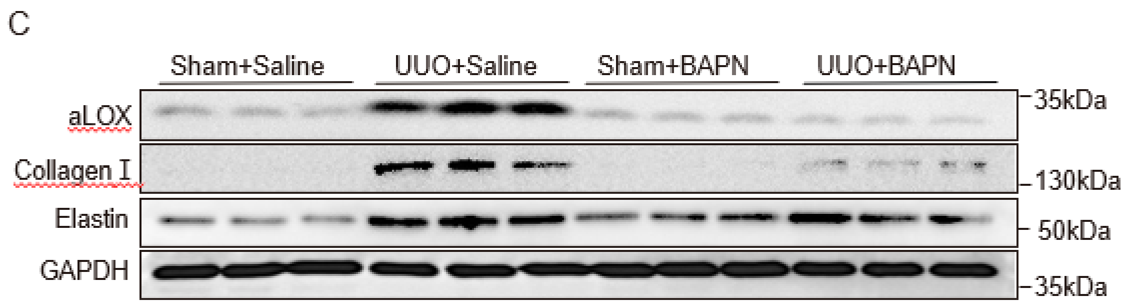

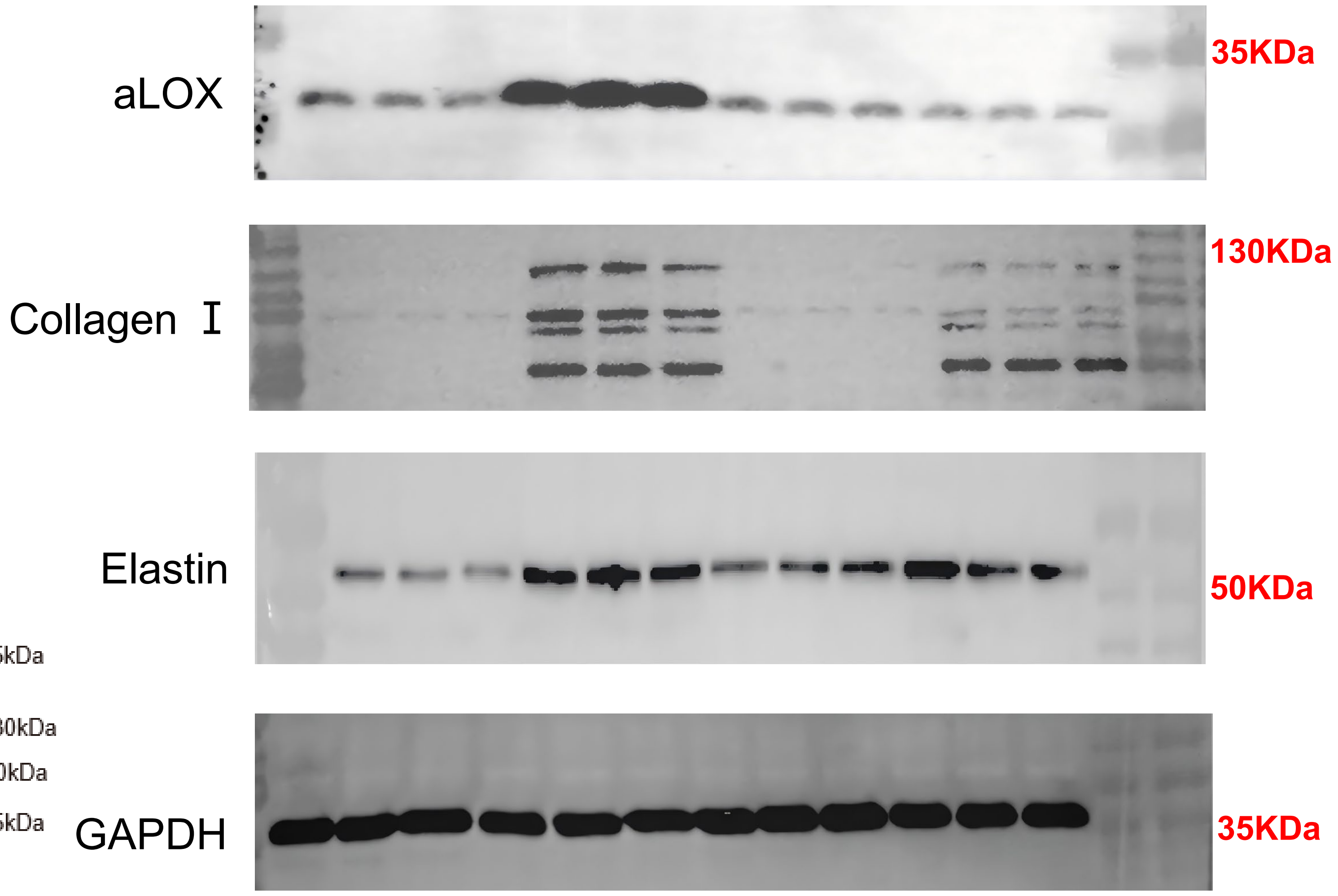

Supplement: Unedited blot and gel images [file jciinsight-11-199028-s154.pdf]
